# Cell Plasticity and Prostate Cancer: The Role of Epithelial–Mesenchymal Transition in Tumor Progression, Invasion, Metastasis and Cancer Therapy Resistance

**DOI:** 10.3390/cancers13112795

**Published:** 2021-06-04

**Authors:** Sofia Papanikolaou, Aikaterini Vourda, Spyros Syggelos, Kostis Gyftopoulos

**Affiliations:** Department of Anatomy, University of Patras School of Medicine, Rion, 26504 Patras, Greece; sof2008_pap@yahoo.gr (S.P.); katevourd@upatras.gr (A.V.); sasyggelos@upatras.gr (S.S.)

**Keywords:** prostate cancer, EMT, metastasis, cell plasticity, therapy resistance

## Abstract

**Simple Summary:**

Although epithelial-to-mesenchymal transition (EMT) is a well-known cellular process involved during normal embryogenesis and wound healing, it also has a dark side; it is a complex process that provides tumor cells with a more aggressive phenotype, facilitating tumor metastasis and even resistance to therapy. This review focuses on the key pathways of EMT in the pathogenesis of prostate cancer and the development of metastases and evasion of currently available treatments.

**Abstract:**

Prostate cancer, the second most common malignancy in men, is characterized by high heterogeneity that poses several therapeutic challenges. Epithelial–mesenchymal transition (EMT) is a dynamic, reversible cellular process which is essential in normal embryonic morphogenesis and wound healing. However, the cellular changes that are induced by EMT suggest that it may also play a central role in tumor progression, invasion, metastasis, and resistance to current therapeutic options. These changes include enhanced motility and loss of cell–cell adhesion that form a more aggressive cellular phenotype. Moreover, the reverse process (MET) is a necessary element of the metastatic tumor process. It is highly probable that this cell plasticity reflects a hybrid state between epithelial and mesenchymal status. In this review, we describe the underlying key mechanisms of the EMT-induced phenotype modulation that contribute to prostate tumor aggressiveness and cancer therapy resistance, in an effort to provide a framework of this complex cellular process.

## 1. Introduction

Prostate cancer (PCa) is the second most frequently diagnosed male malignancy and the second leading cause of cancer mortality in men [1]. As a general rule, both incidence and mortality of PCa correlate with advancing age, with the average age at the time of diagnosis being 66 years [2]. For African American men, the incidence rates are higher when compared to White men; alarmingly, they also have the highest chance of being diagnosed at a younger age (<40 years) [3]. However, this is also true for men generally; the incidence of PCa in young men is rising, and their younger age poses a higher risk for metastatic disease and eventually a higher mortality [3]. It is estimated that mortality will double from 2018 to 2040, reaching 379,005 deaths worldwide. The highest mortality rates are expected in Africa (+124.4%) and Asia (116.7%), while the lowest incidence is expected to be registered in Europe (+58.3%) [4]. It is well known that PCa is a multifocal disease with a heterogeneous cell population. This excessive heterogeneity and plasticity which characterize prostate tumors underline the regulatory phenotypic changes in individual cells that contribute to metastasis and therapeutic resistance [5]. The critical problem in the available pharmaceutical therapeutic approach of PCa is that PCa unavoidably develops resistance to androgen deprivation therapy (ADT) at some stage and progresses to castration-resistant PCa (CRPC), which is characterized by invasiveness and metastatic spread of CRPC cells; this phenomenon still represents the major cause of PCa-related death. Thus, understanding the cellular and molecular mechanisms underlying the process of metastatic dissemination of PCa remains a challenge for better management of PCa.

**Figure 1:**
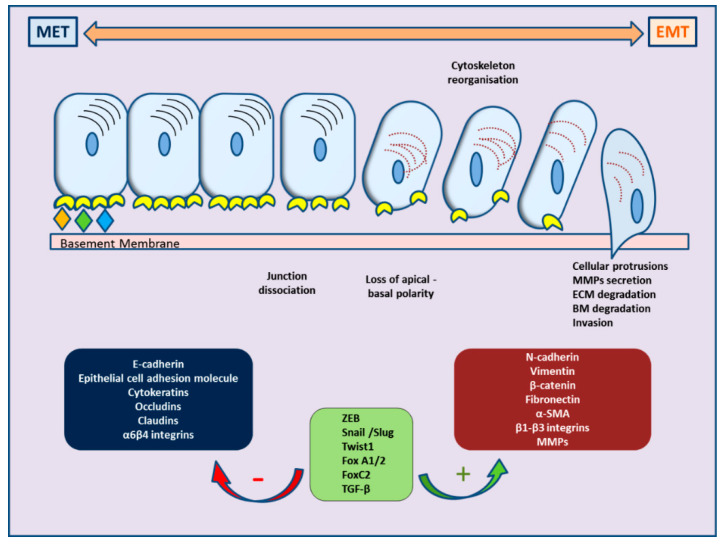
Schematic representation of cellular changes and main molecular events during epithelial–mesenchymal transition (EMT) and the reverse MET.

Recently, ample evidence of epithelial–mesenchymal plasticity playing a role in both PCa metastatic progression and treatment resistance has been provided in several reports. Epithelial–mesenchymal transition (EMT) is a molecular cellular program essential for development, and the relevant processes are reactivated during wound healing, fibrosis, and cancer progression [6,7]. During EMT, epithelial cells lose their junctions and apical–basal polarity, restructure their cytoskeleton, undergo changes in the signaling programs that specify cell shape, and reprogram gene expression; this enhances the motility of individual cells and empowers the progression of a more invasive phenotype [8,9]. EMT is identified by a loss of epithelial markers such as cytokeratins and E-cadherin, followed by a concurrent increase in mesenchymal markers such as N-cadherin and vimentin [10]. In cancer progression, this is connected with poor clinical outcome and therapy resistance in several cancer types [11]. In PCa, for instance, loss of E-cadherin expression and overexpression of N-cadherin correlate with tumor grade and recurrence after surgery, providing a clinically significant link of EMT to aggressive clinical behavior in advanced disease [12]. The cellular processes of EMT are coordinated by several key transcription factors (e.g., TWIST, SNAI1, SNAI2, ZEB 1/2) that act in unison with several epigenetic mechanisms and post-translational protein modifications to arrange the cellular alterations [13]. The main molecular events are depicted in Figure 1.

Different critical signaling pathways including TGF-β, WNT, NOTCH, and growth factors are involved in inducing EMT under specific physiological conditions. Extensive research and available data are suggesting that EMT facilitates tumor progression and metastasis in several cancer types. In this review, we summarize and refer to the main molecular events that control the EMT phenomenon in PCa, in order to better describe its role and mechanisms in PCa progression and therapeutic resistance.

## 2. Molecular Events Governing EMT in Cancer Progression

Epithelial–mesenchymal plasticity has drawn particular interest in the field of research due to its crucial role in facilitating metastases, stemness, and immune repression. EMT is a multi-faceted program which comprises alterations in mRNA and protein abundance, protein modification, and relocation, which, in turn, govern changes in cell morphology, cellular junctions, and apico-basal polarity, resulting in invasive and migratory properties of the cell [14]. Key regulators, including SNAIL, TWIST, and zinc-finger E-box-binding (ZEB) transcription factors, regulate the changes in gene expression that contribute to the repression of the epithelial phenotype and activation of the mesenchymal phenotype. Their expression is an early event in EMT, suggesting key roles in development, fibrosis, and cancer [15]. These transcription factors have diverse expression profiles, and their contribution to EMT is cell/tissue type-dependent. They often regulate the expression of each other, but they may also work together at target genes; during this process, additional transcription factors further fine-tune the EMT transcription program and drive EMT progression [16]. In general, the EMT transcription factors organize the induction of mesenchymal genes and the repression of epithelial genes; frequently, the same transcription factors direct both repression and activation [6].

Although the driving forces of EMT and its reverse process, mesenchymal-to-epithelial transition (MET), have long been identified in developmental processes, they have rather recently received attention in the context of cancer. In several studies regarding cancer progression, EMT has been linked with increased metastatic potential, drug resistance, immune evasion, poor prognosis, and decreased patient survival [17,18,19,20,21].

Of particular interest is the available research focusing on the role of EMΤ in metastasis. The EMT phenomenon is characterized by cellular plasticity. The totally reverse phenomenon to EMT, namely, MET (mesenchymal–epithelial transition), is essential for regrowing at metastatic sites [22]. It has been postulated that the tumor cells undergo MET, a process reverse to the initial EMT, through re-expression of epithelial markers and downregulation of mesenchyme markers, which in turn allows for tumor cell adhesion and colony formation at the new site. This phenomenon was inconsistent with the initial EMT theory, and the reverse process (MET) was considered as a necessary element of the metastatic tumor process [23]. Consequently, the cells enter the invasion phase, by invading the basal membrane and travel through the blood and lymphatic stream (the stage of intravasation). Adjacent tissues may also be invaded during this process. Cells with mesenchymal phenotype may exit the vessels (extravasation) and attach to other organs (secondary sites). These three consecutive stages are not a standard route of all tumor cells, but those cells that manage to “establish” themselves at the secondary sites can proliferate in this new microenvironment, reaching the stage of colonization and forming micrometastases, which in turn may give rise to a secondary tumor [24]. In many solid tumors (for instance, colorectal, breast, prostate, and lung cancer), cells from the secondary tumor site were found to be less differentiated than their primary counterparts, and cells from the primary and secondary tumors share a similar epithelial profile [24]. Recently, the concept of hybrid epithelial/mesenchymal state has been introduced in many primary human cancers including PCa, in order to explain the complexity of changes in cell phenotype that do not comply with a binary status of epithelial or mesenchymal morphology [25]. This concept is supported by relevant data from observations in cell lines in vitro, as well as by the existence of a hybrid phenotype in circulating tumor cells (CTCs) [25,26]. Thus, the concept of EMT as a “spectrum” rather than a binary status more accurately depicts the cell plasticity during cancer progression and metastasis.

### 2.1. EMT and Signaling Pathways: The Crosstalk with Prostate Cancer

**Figure 2:**
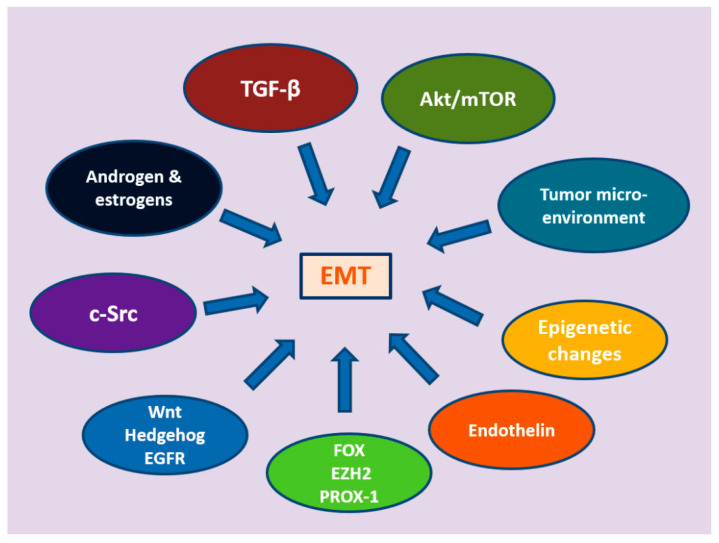
The multiple signaling pathways mediating epithelial-to-mesenchymal transition.

EMT is a multidimensional process, governed by multiple pathways that range from well-established growth factors to hormones, epigenetic changes, and alterations at the tumor microenvironment (Figure 2).

#### Transforming Growth Factor-β (TGF-β): A Signaling Master of EMT

TGF-β (transforming growth factor β) represents one of the key players in the induction of the EMT process. Although its role in many tumors types is multifunctional, in this case, it acts as a tumor suppressor by inhibiting cell proliferation through the repression of c-Myc and certain cyclin-dependent kinase inhibitors (CDKIs) and through the release of antiangiogenic factors [27,28,29,30,31]. TGF-β signaling is mediated through Smad and non-Smad pathways, which in turn are transduced by TGF-β ligands, type 1 and type 2 receptors, and Smad or non-Smad proteins, including Akt, extracellular signal-regulated kinase (ERK1/2), and p38 mitogen-activated protein kinase (MAPK). There are three types of TGF-β in mammals (TGF-β1, 2, and 3), which are encoded by different genes and function through the same receptor signaling systems. TGF-β1 is the one most frequently upregulated in tumor cells [32,33,34].

Three TGF-β ligands (T TGF-β1-3) and the relative TGF-β type 1 (TGF-βR1) and type 2 receptors (TGF-βR2) are basically responsible for TGF signaling [21,22,23]. TGF-βR1 recruits and phosphorylates receptor-regulated Smads (R-Smads). TGF-β ligands can bind with high affinity to T TGF-βR2 after activation by several metalloproteinases (MMPs). This binding leads to the formation of a heterotetrameric active receptor complex, which marks the phosphorylation of TGF-βR1 by T TGF-βR2. R-Smads may also become phosphorylated by TGF-βR1 and subsequently form heteromeric complexes with co-Smad (Smad4 in mammals); translocation of these R-Smad/co-Smad complexes into the nucleus regulates the expression of diverse target genes with other DNA-binding transcription factors [35,36,37].

Furthermore, TGF-β is able to activate “noncanonical” signaling pathways, often called non-Smad pathways. The ERK/MAPK pathway and the phosphoinositide 3-kinase (PI3K)/Akt pathway activation by TGF-β1 are two well-known examples [38,39]. These noncanonical Smad pathways may perform either separately or together with Smad complexes, for example, through inhibition of canonical Smad signaling through R-Smad sequestration [40]. Interestingly, the widely used cholesterol-lowering drug simvastatin has been shown in vitro to attenuate TGF-β1-induced EMT, cell migration, and invasion in DU145 cells, through a non-Smad pathway [41]. A similar effect of simvastatin has been shown on tumor-associated macrophages and tumor cells in a non-small-cell lung cancer (NSCLC) cell line; simvastatin attenuated TGF-β, which, in turn, remodeled the tumor microenvironment and suppressed EMT [42].

Additionally, TGF-β receptors may activate MMPs and p38 MAPK through non-Smad pathways and induce the expression of transcription factors SNAIL, SLUG, ZEB1, and ZEB2, via the Smad pathway, which both lead to EMT. TGF-β-induced EMT has been implicated in benign prostatic hyperplasia (BPH), where stromal cells may induce EMT, via TGF-β1 secretion and signal activation. In advanced stages of PCa, TGF-β has been found to be upregulated, contributing to increased cell invasion and metastasis [43]. Stromal fibroblasts promote PCa by modulating the adjacent epithelial cell growth and its oncogenic potential through TGF-β pathways [44]. Additionally, in PCa cell lines, TGF-β has been shown to guide the nuclear accumulation of nuclear factor-kappa B (NF-κB), a probable intermediate for the acquisition of a mesenchymal phenotype. Importantly, inhibition of NF-κB seems to reverse the EMT process by revoking vimentin activity [45,46].

The type III TGF-β receptor (TβRIII or T TGF-βR3, also known as betaglycan) is a coreceptor that is not involved directly in TGF-β signal transduction; rather, it mediates TGF-β superfamily signaling to both Smad and non-Smad signaling pathways. Loss of T TGF-βR3 expression has been noted during cancer progression. Thus, TGF-βR3 is likely a suppressor of cancer progression and metastasis, as it appears to exert direct effects on cell proliferation, migration, invasion, and neoangiogenesis. Restoration of TGF-βR3 expression in human PCa cells may lead to inhibition of migration and invasion, independently of the ligand TGF- β. In a human PCa xenograft model, restoring TGF-βR3 function decreased tumor growth, suggesting its tumor suppressor role [47].

### 2.2. TGF-β Signaling and Androgen Axis in Prostate Cancer

In PCa, expression of vimentin induced by TGF-β is associated with biochemical recurrence, and TGF-β from bone marrow-derived mesenchymal stem cells promotes metastasis of PCa cells [46,48]. Notably, upregulation of TGF-β3 has been shown to strongly affect migration and invasion by PCa cells. Such results have raised great interest in therapeutic targeting of this pathway in advanced PCa, and anti—TGF-β therapies are progressing [49,50].

The TGF-β signaling cascade plays a dual role in PCa; in early tumor stages, it acts favoring proliferation arrest and apoptosis, in synergy with androgens. However, it apparently promotes progression to metastasis in advanced PCa disease [47]. The inhibitory action of the androgen receptor (AR) on TGF β1-induced transcriptional activity in PCa cells is enhanced by an AR coactivator, AR-associated protein 55 (ARA55). In a study of human hepatoma cells, a similar interaction of TGF-β1 transcription with androgens and the AR complex has also been shown [51]. However, the interaction of AR and TGF-β1 is dynamic and multifaceted. Data from studies on PC-3 and LNCaP human PCa cell lines show that SMAD4 (either alone or in the form of a SMAD3/4 complex) interacts with the AR transcriptional activation domain, controlling the DHT-induced transcriptional activity of the androgen receptor [47]. In an androgen-independent PC-3 model, forced expression of AR resulted in inhibition of the transcriptional activity of TGF-β1/SMAD, along with TGF-β1-induced growth inhibition and apoptosis [47]. Inhibition of the tumor suppressor function of TGF-β in PCa cells is probably mediated via suppression of SMAD3 induced by AR activity [52,53]. Earlier findings indicate that the AR negatively regulates the expression of TGF-β via a negative androgen-response region composed of multiple androgen-response elements (nAREs) [54]. However, beyond the coaction with TGF-β, androgen (and other steroids) signaling is per se an important pathway in epithelial–mesenchymal transition in PCa.

### 2.3. Androgen and Estrogen Signaling in EMT

The prostate is an established androgen-dependent gland: its embryonic development and normal function are guided by the androgen/AR signaling cascade [55]. One central characteristic of PCa oncogenesis and disease progression is the action of androgens and AR signaling, as the androgenic action of dihydrotestosterone (DHT)—in particular—promotes survival in prostatic epithelial cells. The fact that prostatic cancer cells are androgen-dependent to advance cell growth and inhibit apoptosis has led to the acceptance of ADT as the mainstay of pharmaceutical treatment, especially for metastatic disease; however, almost all tumors will become castration-resistant with time [56]. Clinical and biochemical exacerbation occurs in an androgen-independent manner and is associated with poor prognosis. EMT seems to play a significant role in the progress of metastatic CRPC. Preclinical models of CRPC have highlighted the relationship between the androgen axis and distinct pathways regulating apoptosis, EMT, and invasion, providing evidence that EMT is controlled by the androgen signaling axis, affecting tumor progression, metastasis, and resistance to therapy [47,54,57].

Androgen-mediated activation of β-catenin may be a process via which the androgenic axis induces EMT of prostate tumor epithelial cells [58]. AR function as a transcriptional repressor of E-cadherin has lately been compared to that of Snail and Twist. Activated AR has been shown to be able to suppress E-cadherin gene expression in breast cancer and metastatic prostate cell lines, attributed to a mesenchymal phenotype [57,59].

Recently, BMI1 (B lymphoma Mo-MLV insertion region 1) has been featured as a direct target gene of AR. BMI1 represses tumor suppressors and promotes cell proliferation and cancer progression, possibly through EMT. It has been shown that DHT upregulates both mRNA and protein levels of BMI1, and that BMI1 increases in CRPC in a mouse xenograft model [60]. Interestingly, the EMT pattern in PCa cells can be induced by androgens, bypassing the functional involvement of TGF-β; this is mediated through the activation of the transcription factor Snail, leading to an enhanced PCa cell migration and invasion potential and aggressive metastatic behavior [58].

However, the effect of androgen deprivation seems to have a dark side; although the mechanisms underlying castration resistance are not fully understood, it has become apparent that pharmaceutical castration itself may be responsible for the development of EMT. Following androgen deprivation, prostate tumor cells exhibit EMT and increased stem-cell-like features, a procedure attributed to a feedback loop involving the androgen receptor and the Zeb1 transcription factor [61]. Similarly, overexpression of N-cadherin in PCa cells may further provoke EMT, invasion, and metastasis; expression of N-cadherin is increased after androgen deprivation, and its aberrant expression is involved in metastatic CRPC [62,63]. Interestingly, in this type of tumor, increased levels of N-cadherin are mainly found in poorly differentiated areas and significantly correlate with advanced Gleason grade [64,65]. Overexpression of N-cadherin reduces intracellular adhesiveness and may also guide stromal–epithelial interactions (between stromal fibroblasts and tumor epithelial cells), eventually promoting cell motility, invasion, and metastasis. Of clinical importance, increased N-cadherin expression has been found to be a meaningful predictor of clinical recurrence in PCa patients subjected to radical prostatectomy [64,65,66]. Another pathway for the ADT-induced EMT is mediated through the chemokine C–C motif ligand 2 (CCL2), important in recruiting tumor-associated macrophages. ADT reduces a transcriptional repressor (SAM pointed domain-containing ETS transcription factor, SPDEF) of CCL2, which mediates EMT of prostate tumor cells. In tissues from PCa patients receiving ADT, low SPDEF levels were correlated with high CCL2 expression compared to treatment-naïve patients [67].

It is also worth mentioning that androgen deprivation therapy may also lead to neuroendocrine differentiation (NED) of the tumor cells, a status associated with PCa progression to a hormone-refractory state and poor prognosis. During this trans-differentiation, PCa cells shift to neuroendocrine-like cells and express typical neuroendocrine markers (e.g., chromogranin, synaptophysin, neuron specific enolase (NSE), and CD56). These neuroendocrine cells secrete factors (neuropeptides and growth factors) in a paracrine fashion; these factors act as a mitogenic stimulus on neighboring cells and promote androgen-independent growth of PCa cells. This also affects stromal cells, distressing the entire tumor microenvironment [68]. Although the development of NED is considered to occur mainly through stem-cell differentiation and trans-differentiation under ADT, a possible role for EMT linking still is under investigation [69]. A possible link has recently been suggested through the AR antagonist effect of alleviating the normal repression that AR exerts on SPINK1 (serine peptidase inhibitor, Kazal type 1), a critical player for maintenance of the NE phenotype. ADT leads to SPINK1 upregulation through increased SOX2 expression, and the transactivation of SPINK1 elicits epithelial–mesenchymal transition, stemness, and cellular plasticity [70]. Another possible pathway includes the signaling cascade of the Wnt/β-catenin pathway, as discussed below [71].

On the other hand, the effect of estrogens and their receptors on PCa biology remains obscure, despite increased interest in their role in prostate tumorigenesis and PCa progression. Estrogen receptor-α (ER-α) is primarily expressed in basal cells and prostatic stroma, whereas estrogen receptor-β (ER-β) expression is higher in the glandular epithelium, where ER-β is accountable for regulating its proliferation and differentiation [72].

It has been long accepted that ER-α mediates cell survival and proliferation induced by estrogens, while ER-β acts as a tumor suppressor, facilitating a protective and antiapoptotic effect of estrogens in PCa [73]. However, the available data on the balance between the two receptors in different stages of PCa progression are confusing. Older studies verified the presence—albeit limited—of ER-α in prostate carcinoma samples, in both stroma and glandular epithelium; however, no correlation with advanced or high-grade tumors has been found [74]. In contrast, increased ER-α gene expression on mRNA and protein levels was observed in hormone-refractory tumors and metastatic lesions, including lymph node and bone metastases [75]. The re-emergence of the ER-α and ER-α-regulated genes and the partial loss of ER-β during PCa progression and hormone-refractory disease suggest a bypass of the androgenic cascade for these tumors and the utilization of alternative steroidal pathways [76].

Interestingly, these changes in ER content may lead to EMT changes through several pathways. The aberrant increase in ER-α may lead to EMT changes through transcriptional regulation of the nuclear-enriched abundant transcript 1 (NEAT1), a significantly overexpressed long non-coding RNA (lncRNA) associated with PCa progression [77]. An alternative pathway described is the ER-α driven overexpression of neurogenic locus notch homolog protein 1 (NOTCH1) [78]. The “hypoxia-driven” hypothesis has been proposed for ER-β, the ERβ1 subtype in particular, suggesting suppression of ERβ1 and stimulation of HIF-1α-mediated VEGF-A expression as the main EMT-inducing event [73]. At the other end of the spectrum, the suppression of the “protective” ERβ1 may be corroborated by the action of the ERβ2 and ERβ5 receptor subtypes, either through stabilization of HIF-1α and further expression of hypoxic genes or through ERβ2-mediated overexpression of Twist1 and Slug [79,80]. Lately, the role of estrogen-binding sites at the plasma membrane (PM) level has been proposed as an alternative signaling pathway that contributes to higher invasiveness in PCa cells. Common EMT features (for example, increased migration and invasion, higher vimentin, and decreased E-cadherin expression) have been induced in LNCaP cells by cell-impermeable estradiol (E2-BSA) that acts at the PM level [81].

There is cumulative evidence that both members of the steroid superfamily (androgens and estrogens) may interact and drive significant EMT changes in PCa cells, accounting for more aggressive phenotypes, advanced disease, and hormone-refractory tumors. However, the complex pathways are still not clearly comprehended. More in vivo studies are probably needed, as the commonly used PCa models are likely inappropriate to accurately study the steroid receptors and their action or inhibition [82].

### 2.4. Src Signaling

It is well accepted that c-Src, a non-receptor tyrosine kinase, regulates a complex signaling network facilitating the development of castration-resistant PCa and bone metastases [83,84,85,86,87,88]. Many preclinical studies have underlined the role of c-Src and Src family kinases (SFKs) in proliferation, angiogenesis, invasion, and bone metabolism, contributing to disease progression through mechanisms at the epithelial and stromal compartment [83,84,85,86,87,88]. It has been shown that Src inhibitors decrease the proliferation, invasion, and migration of PCa cell lines in vitro and, similarly, Src inhibition represses androgen-independent growth and metastasis [89]. Src-family (SFK) and Ack1 non-receptor tyrosine kinases play a significant role in activating AR through direct phosphorylation, causing a response to very low levels of tissue-expressed, so-called intracrine, androgens, thus facilitating pathways that mediate PCa proliferation, anti-apoptosis, and oncogenic aggressiveness [90]. Hypoxia may be a key feature that enhances metastatic-associated cell functions by activating c-Src in PCa cells [91]. Interestingly, the tumor microenvironment seems to contribute to Src signaling and EMT triggering via myofibroblasts and neutrophil-derived specific proteins. The stromal myofibroblasts secretory chemokine CXCL1 and neutrophil-derived cytokine LCN2 have been found to act as a paracrine network that promotes migration of PCa cells, leading to enhanced tumor metastasis [92]. Another non-receptor tyrosine kinase related to SRC family kinases, protein tyrosine kinase 6 (PTK6), has been found to enhance the epithelial–mesenchymal transition (EMT) by inhibiting E-cadherin expression and inducing expression of the mesenchymal markers vimentin, SLUG, and ZEB1 [93]. Parallel to Src, the androgen receptor may interact with p85a, the regulatory subunit of PI3K, a recognized oncogenic protein kinase with a key role in malignant transformation, metastasis, and progression of the disease [94,95].

### 2.5. Wnt, Hedgehog, Notch Signaling, and EGFR Signaling

WNT signaling, the binding of WNT ligands to Frizzled protein receptors, results in the inhibition of GSK3β, thus preventing phosphorylation, ubiquitylation, and degradation of β-catenin, allowing for regulation of gene expression. Inhibition of the GSK3β kinase increases SNAIL stability, thus promoting EMT [96,97,98]. WNT signaling plays several roles in EMT during embryonic development [97]. Canonical WNT signaling also enables the generation and proliferation of neural crest precursors, whereas GSK3β-independent WNT signaling is necessary for the migration of neural crest cells. WNT signaling also promotes cancer progression-associated EMT, mainly at the invasive front of different tumors, where EMT occurs [6,98,99,100,101]. The Wnt pathway has been found to affect prostate tumorigenesis through androgenic signaling. Hence, mechanisms of the Wnt signaling pathway participate in crosstalk with the androgenic/AR signaling axis and are able to regulate the AR transcriptional activity [102]. Several Wnt factors (for example, β-catenin, glycogen synthase kinase (GSK3)β, the lymphoid enhancer binding factor 1 (LEF1), and cyclin D1) have been found to interact with the androgen axis [103,104]. In the cytosol, β-catenin is attached to a multiprotein complex; β-catenin cytosolic accumulation may occur when Wnt signaling is activated. This is affected by inhibition of the (normally occurring) GSK3β-targeted proteosomal degradation of the β-catenin/multiprotein complex. Translocation of accumulated β-catenin into the nucleus results in coactivation of target genes, such as the T-cell factor/lymphocyte-enhancer factor (TCF/LEF) and AR genes [104,105,106,107]. Nuclear β-catenin signaling is associated with PCa progression; notably, reduced nuclear expression of β-catenin in higher-Gleason-grade prostate carcinoma with increased has also been reported [107,108].

Aberrant activation of the Wnt/β-catenin pathway correlates with EMT features and may also be involved in regulating stemness of the PCa stem cells (PCSCs) in PCa. In a recent study, low levels of the circadian rhythm gene PER3 (period circadian regulator 3) were associated with increased stemness of PCSCs, possibly mediated through phosphorylation of β-catenin and the activation of the WNT/β-catenin pathway [109]. Similarly, miR-320, a CD44^+^ PCSC negative regulator, has been shown to reduce β-catenin expression and repress CSCs via Wnt/β-catenin pathway inhibition. In addition, some PCSC features (e.g., sphere- and colony-forming abilities, chemoresistance) are enhanced by downregulation of miR-320 [110].

Another pathway involved is Hedgehog (HH) signaling, an important pathway during embryonic development of almost all organs in mammals, where ligand binding to transmembrane protein receptors (patched receptors—PTCs) activates transcription factors of the glioma (GLI) family [111]. During normal development, Sonic HH (SHH) induces the expression of sclerotomal cell markers in cells that transit into the mesenchyme to form the sclerotome [112]. The ectopic expression of GLI1 in rat kidney epithelial cells has been shown to induce SNAIL1 expression and loss of E-cadherin, as well as increased SHH expression and signaling, features that are associated with increased SNAIL1 expression in epithelial cancers [113,114,115,116].

In the context of tumorigenesis, the HH pathway may be considered an “autocrine loop”, since the tumor cell produces the HH ligands and causes cell-autonomous HH pathway activation [115]. There is also a “paracrine loop”, however, as the HH pathway has often been present in the surrounding stromal elements in several tumors, including PCa [117,118,119]. Thus, tumor cells produce HH ligands and signals toward the tumor microenvironment, i.e., the surrounding stroma, which in turn facilitates tumor progression. Following observations in B-cell malignancies, another type of paracrine signaling called “reverse paracrine signaling” has also been described; in this case, HH ligands are secreted from the stroma leading to stimulation of tumor survival and growth [120]. It is still unclear whether the mode of aberrant HH signaling in PCa is purely autocrine or paracrine; nevertheless, there is cumulative evidence on the dynamic role of HH signaling in the development and progression of PCa [121,122]. Generally, aberrant HH signaling in prostate tumors is believed to be ligand-dependent, in autocrine or paracrine mode or both. However, activation of the HH pathway seems more prominent in advanced PCa. A correlation with advanced tumor grade and stage has been shown in several studies [121,122,123,124,125]. Moreover, increased expression of HH signaling components has been found in metastatic PCa samples [121,122,123,124,125].

The crucial role for HH signaling in the progression to androgen-independent PCa has also drawn attention recently. Data from studies with both PCa cell lines and tumor specimens have highlighted the possible detrimental effect of long-term ADT, which was shown to induce an upregulation of HH signaling [125,126]. This phenomenon may be partly explained by a paracrine mode of HH signaling through the tumor microenvironment, which results in acquired intratumoral steroidogenesis [127]. Interestingly, in vitro studies utilizing PCa cells indicate that HH inhibition results in suppression of EMT. In a recent study, the newer Shh inhibitor vismodegib significantly inhibited EMT in CRPC cells and tumor growth in a mouse model [128].

Notch signaling, one of the oldest identified signal transduction systems, is part of EMT during embryogenesis and potentially in EMT during cancer progression. Activation of Notch signaling usually starts by binding of the ligand expressed on one cell to the receptors expressed on its adjacent cells. This is followed by two sequential proteolytic cleavages of the receptors and the release of the Notch intracellular domain, which subsequently translocates into the nucleus and regulates target gene expression [129]. During development, Notch signaling is required in virtually all developing systems. However, Notch signaling is also involved in tumorigenesis; emerging evidence has suggested that the Notch pathway regulates cell growth, apoptosis, cell cycle, and metastasis. Data from available studies imply that Notch receptors have a dual nature, acting either as oncoproteins or tumor suppressors in several types of human cancers, including PCa [130].

Components of the Notch signaling cascade are normally expressed in prostate epithelia, suggesting that Notch plays a crucial role in prostate development and homeostasis [131]. Notch, however, is also a potential driver in PCa development. Overexpression of Notch-1 was found to contribute to migratory activity, as well as cell detachment and attachment of PC-3 cells; this also induced EMT changes and exhibited cancer stem cell features in Pca cells [132]. Moreover, Jag1 and Notch1 expression has been found elevated in high-grade and metastatic PCas [133]. Along these lines, Notch-4 silencing suppressed the viability and proliferation in the PCa cell lines DU145 and PC3 and resulted in decreased cell migration and invasion, affecting the expression of EMT markers, possibly via the NF-κB pathway [134]. These findings suggest that the Notch signaling pathway may serve as a potentially useful therapeutic target.

Epidermal growth factor receptor (EGFR), also known as HER1 or ERBB1, is essential for the development and progression of various cancers, including colorectal, head and neck, gastric, non-small-cell lung, and PCa [135,136,137] Hence, EGFR seems a promising therapeutic target, with the development of specific anticancer drugs such as EGFR-tyrosine kinase inhibitors (TKI). EGFR, a member of the transmembrane receptor tyrosine kinase family is activated after binding of several ligands, including EGF and TNFα. Ligand binding leads to the formation of active homodimers (or heterodimers) and the transphosphorylation of EGFRs. Subsequent activation of SRC homology 2 (SH2) domain-containing downstream signaling proteins initiates several signal transduction cascades and further regulates their downstream effectors, including mitogen-activated protein kinase (MAPK), JNK, and phosphoinositide-3-kinase (PI3K)/AKT pathways. These in turn control cell-cycle progression, cell survival, and invasion [138]. High levels of nuclear EGFR have been found in various types of cancers [139,140], and nuclear EGFR signaling has been involved in acquired radiation resistance and chemoresistance to cisplatin [141,142,143,144]. In the normal prostate, expression of EGFR is low compared to primary and metastatic PCa tissues. Both EGF and EGFR are expressed in androgen-independent and metastatic PCa, which often present with a mesenchymal phenotype. This correlates with higher Gleason score, increased metastatic potential, and an overall poor clinical prognosis and reduced survival rate [145,146,147]. Other studies have also shown that aberrant activity of EGFR may facilitate the development of CRPC, possibly due to the interruption of androgen signaling [148]. Recently, the proven ability of tumor cells to use extracellular vesicles (EVs) as a mean to disseminate EGFR and ligands to both neighboring and distant cells is under investigation for its diagnostic, prognostic, or therapeutic potential [149].

Spautin-1, an inhibitor of ubiquitin-specific peptidase 10 (USP10) and USP13, has been recognized as an autophagy inhibitor [150]. Further studies showed that Spautin-1 enhances the antitumoral activity of targeted therapies or chemotherapy via inhibition of autophagy in diverse tumors [150,151,152,153]. In a recent study, Spautin-1 was found to inhibit EGFR signaling and suppress the growth of PCa [150]. The Spautin-1-mediated inhibition of EGFR further inactivates the MEK/ERK/cyclin D1 axis and decreases Glut1 expression, while activating the MKK4/JNK/Bax axis, which induce cell-cycle arrest and apoptosis of PCa cells. Moreover, the use of Spautin-1 improved the anticancer activity of the androgen receptor inhibitor enzalutamide both in vitro and in vivo [150]. Keeping in mind that EGF-mediated EMT is a common feature in several cancers, the use of Spautin-1 as an EGFR signaling inhibitor may prove a promising novel addition to therapeutic schemes for several cancers, including PCa.

### 2.6. Novel Transcription Factors Regulating EMT in PCa

The Forkhead box (FOX) family includes several transcription factors displaying a conserved winged-helix DNA-binding domain (DBD) or “forkhead” domain. This DBD is common to all FOX family members; however, distinct transactivation and repression domains exist between different tissue and cell-type transcription factors. In general, FOX transcription factors are considered to play an important role during embryogenesis and cellular homeostasis [154,155]. Both Forkhead box protein FOXC1 and FOXC2 transcription factors are implicated in cancer progression, by driving angiogenesis, invasion, and metastasis. In patients with breast and hepatocellular cancer, FOXC1 has been found to positively correlate with tumor metastasis and poorer prognosis [154]. FOXC2 is—among other tumors—overexpressed in PCa [154]. Forkhead box protein C2 (FOXC2) acts as an epithelial-to-mesenchymal transition (EMT)-regulating transcription factor, through its interaction with the cadherin family, kinases, and other regulators. Several studies underline the role of FOXC2-induced EMT, either through inhibition of the expression of E-cadherin in the adhesion part of epithelial cells or through activation of the Akt pathway and increased expression of Snail and p-(glycogen synthase kinase 3β) GSK-3β [155,156,157]. In an immunohistochemical study of prostate carcinoma specimens, increased FOXC2 expression and EMT phenotypes were associated with castration resistance, metastasism and poor survival in PCa cases [158]. Interestingly, inhibition of FOXC2 by a p38 inhibitor restores epithelial cell characteristics and drug sensitivity in PCa cells with stem-cell properties that are insensitive to ADT [159]. The central role of upregulated FOXC2 by various EMT inducers, along with the ability to block ‘targetable’ regulators of FOXC2 (such as the p38 MAPK) by small molecules, may prove a promising approach.

However, beyond FOXC transcription factors, a variety of other Forkhead box proteins have recently been the focus of research regarding their key role in EMT and cancer progression. FOXA1 is considered a pioneer factor of PCa onset and progression, through induction of open chromatin conformations that allow binding of several transcription factors, mainly the AR in both normal and cancerous prostate cells [160]. Another AR-independent pathway allows coding and noncoding mutations of FOXA1 to promote EMT. Although direct inhibition of transcription factors such as FOXA1 is difficult, targeting their EMT regulatory pathway may be a promising strategy, as seen in preclinical studies [49,161]. Similarly, the FOXO protein members have been implicated as important modulators of EMT in several cancers [162]. In PCa cell models, the Nodal pathway leads to EMT changes and metastasis through FOXO1/3a activation; interestingly, treatment of PCa cells with FOXO inhibitor AS1842856 resulted in blockage of the Nodal pathway, suggesting a potential target for the control of metastatic disease in PCa cases [163].

The enhancer of zeste homolog 2 (EZH2) is a histone methyltransferase (HMT), acting as the catalytic subunit of PRC2 (polycomb repressive complex 2), a member of the polycomb group proteins that primarily methylate histone H3 on lysine 27, inducing transcriptional silencing of target genes [164]. Aberrant expression of EZH2 has been linked to cancer development and progression in several tumor types, while available data suggest a link with EMT phenotypic changes. EZH2 induces epithelial–mesenchymal transition and the pluripotent phenotype of gastric cancer cells through PTEN/Akt signaling, by binding to a PTEN promoter [165]. In melanoma, an actionable axis linking NFATc2 to EZH2 was shown to control the EMT-like program of melanoma cells, while inhibition of EZH2 downregulated EMT-related gene expression [166]. In several tumors, including PCa, EZH2 overexpression was found to positively correlate with disease progression and poorer prognosis [164].

In PCa, EZH2 expression is significantly elevated and is associated with increased proliferation activity and metastatic capability [167]. As a potential biomarker, EZH2 expression in diagnostic prostate biopsies has been found to be an independent predictor of outcome patients with PCa [167]. Elevated EZH2 expression is also correlated with development of CRPC and even neuroendocrine differentiation, but the mechanisms via which EZH2 enhances PCa progression are still unclear [168,169]. A possible explanation would be the methylation of the AR to regulate specific gene expression, offering survival signals to CRPC cells. However, EZH2 appears to act as a multifaceted transcription factor, with additional polycomb- and methylation-independent roles, as EZH2 also activates AR gene transcription through direct occupancy at its promoter [170].

Strong links between EZH2 and EMT-mediated cell plasticity have been lately highlighted in PCa. EZH2 binds the hepatocyte nuclear factor 1β (HNF1B) locus and suppresses its expression in PCa cell lines; consistently, a reverse correlation between EZH2 and HNF1B expression is present in clinical samples [171]. Thus, HNF1B appears as a direct downstream target of EZH2, and its tumor suppressor role is mediated by repression of SLUG expression and EMT process [171]. Other pathways linking EZH2 and EMT described in other tumor types, such as the Sox4/Ezh2 axis in pancreatic cancer or the EZH2-mediated WNT5A silencing in colon cancer, remain to be proven [22,172].

Under these circumstances, inhibition of EZH2 would expectantly suppress PCa cell proliferation and invasion in vivo and in vitro. Indeed, EZH2 inhibition has been shown to restore AR expression and sensitivity to antiandrogen therapy in preclinical models of advanced PCa, suggesting epigenetic reprogramming as a promising approach to bypass antiandrogen therapy resistance [164]. Thus, novel EZH2 inhibitors are being evaluated in patients with mCRPC. EZH2 inhibition/depletion has been shown to enhance the efficacy of enzalutamide in enzalutamide-resistant PCa cells [173]. CPI-1205, a potent, selective, and cofactor-competitive EZH2 inhibitor is currently under clinical evaluation [174]. When used in line with antiandrogen therapy, EZH2 inhibitors may contribute to improved outcomes in both early- and late-stage mCRPC patients, through their antitumoral activity and ability to re-sensitize hormone-refractory Pca. Moreover, targeting Ezh2 could overcome docetaxel resistance in PCa cells, by suppressing Doc-induced cancer stem-cell populations [175].

The mammalian transcription factor Prospero-related homeobox 1 (PROX1) is a member of the homeobox transcription factor family and acts as a crucial regulator in organ development during embryogenesis and lymphangiogenesis. PROX1 has been attributed with both oncogenic and tumor-suppressive functions in many types of human cancers [176]. However, it is well accepted that PROX1 may promote tumor progression by facilitating cancer cell migration, invasion, and metastasis through its role as a key organizer of the lymphatic system [176].

A link between PROX1 and EMT-induced cell plasticity with increased invasive and metastatic potential has been highlighted in several tumors. For instance, PROX1 promotes EMT in colon cancer cells by inhibiting E-cadherin via miR-9 and, additionally, maintains the stability of transcription factor HIF1α protein, which further induces EMT in hepatocellular carcinoma [177,178]. However, the relationship of PROX1 with EMT in the progression of PCa remains unclear. In a study based on different PCa cell lines and knockout mice, PROX1 could be suppressed by DAB2IP, a novel member of the Ras GTPase-activating protein family. DAB2IP regulates EMT and metastasis of PCa through targeting PROX1 transcription and destabilizing HIF1α protein [179].

### 2.7. Endothelin Axis and EMT in Prostate Cancer

The endothelins (ETs) belong to a family of three small (21 amino acid) peptides, ET-1, ET-2, and ET-3, and exert their effects by binding to the cell-surface ET receptors ET-A and ET-B. Both receptors belong to the G-protein-coupled receptor system [180]. Endothelin has an established role as a powerful endogenous vasoconstrictor and is implicated in vascular diseases of several organ systems, including the cardiovascular, respiratory, and urinary systems; however, ET-1 is also a possible key factor for cancer progression in different tumor types. ET receptor activation (and particular ET-A) promotes tumor progression through several mechanisms, including cell proliferation, EMT induction, angiogenesis, and metastatic spread, suggesting that ETA receptor blockade strategies may enrich the armamentarium of cancer treatment [180].

In head and neck cancer, predominantly squamous cell carcinomas (HNSCCs), ET-1 may promote HNSCC progression via several mechanisms, including EMT [181]. Knockdown of ET-B receptor inhibits the progression of triple-negative breast cancer by promoting the mesenchymal-to-epithelial transition (MET) process [182]. In colorectal cancer, endothelin-1/ET-1 receptor signaling and the β-catenin pathway promote the cross-talk with β-catenin signaling through interaction with the signal transducer β-arrestin1 (β-arr1), to sustain stemness and an EMT phenotype [183]. In ovarian cancer, the ET-1/ET-A R autocrine pathway was found to induce an invasive EMT phenotype; common findings included increased expression of β-catenin, Snail, and other mesenchymal markers, as well as downregulation of E-cadherin [184]. However, little is known about the corresponding ET-induced EMT pathways in prostate carcinoma, and available studies are limited. In an immunohistochemical study of PCa tissue samples, increased expression of ET-1 and ET-A (but not ET-B) was associated with lymph node metastasis, advanced stage, and EMT phenotypic changes, along with increased Snail expression [185]. These data, together with results from other solid tumors, make the use of ET axis inhibitors a promising approach toward PCa progression and metastasis. Although, in a recent meta-analysis, the use of ET-A receptor antagonists with or without docetaxel was not proven to offer improvement in survival or disease progression for CRPC, other recent data suggest that ET-A blockade is most efficacious when combined with complete androgen deprivation for the control of bone metastases [186,187].

## 3. Epigenetic Changes and EMT in Prostate Cancer

Epigenetic mechanisms may facilitate the molecular understanding of EMT, as well as broaden the therapeutic spectrum. Several studies show the important role of reversible epigenetic mechanism in cancer progression. The epigenetic mechanisms are biological processes that do not modulate DNA sequences but affect gene expression and subsequently cell fate [188]. Such epigenetic modifications occur in neoplastic cells during the development and progression of cancer. In particular, cancer cells may obtain their invasive and metastatic characteristics through epigenetic alterations, which may involve chromatin remodeling, DNA methylation, histone modifications, and alterations in the expression of microRNAs (miRNAs; small noncoding RNA molecules involved in the post-transcriptional modification of gene expression) might prevent translation or promote mRNA degradation [189]. DNA methylation is one of the most crucial epigenetic regulatory mechanisms influencing gene expression and was the first example of epigenetic modifications studied in human cancer [188,190]. DNA methylation is a chemical reaction via which a methyl (–CH_3_) group is attached to either a cytosine or adenine of DNA molecules. The members of the DNA methyltransferase family, DNMT1, DNMT3A, and DNMT3B, direct these modifications and are considered crucial epigenetic factors due to their effect on gene expression [191]. Heterochromatin undergoes DNA hypermethylation, which usually results in gene suppression, but two specific methylation patterns, permethyl compounding and hypomethylation, are also associated with carcinogenesis. Hypermethylation occurs in CpG dinucleotides and commonly inactivates tumor promoters by repressing genes in cancer cells, whereas DNA hypomethylation leads to chromatin instability, gene inactivation, or maladaptation [192]. Hyper- or hypomethylation can be removed either passively, after several rounds of DNA replication on the daughter strand, or actively, via multiple enzymes such as ten-eleven translocation (TET) family proteins through the base excision repair (BER) pathway [193].

Several studies indicate that DNA methylation changes are frequent events in advanced PCa, with many critical genes being affected by these changes. It is, therefore, possible to use them as molecular biomarkers of PCa progression and response to treatment [194]. Thus, the well-described GSTP1 promoter hypermethylation and secondary silencing is considered an early event modification in prostate carcinogenesis and, therefore, has been suggested as a promising prognostic biomarker [195,196,197]. Other common hypermethylation changes (up to 30% of the CRPCs) include the hypermethylation of the promoter of the AR, resulting in loss of AR expression, whereas the hypermethylation of the p16 tumor suppressor gene has been associated with increased proliferation [198]. Moreover, PTEN silencing is commonly caused by the hypermethylation of the CpG islands in its promoter, facilitating carcinogenesis and disease progression [198,199]. On the other hand, the hypomethylation of certain prometastatic genes such as heparanase and urokinase plasminogen activator (uPA) seems to actively promote tumor invasion and metastasis [200]. Interestingly, concurrent DNA hypo- and hypermethylation patterns may develop under the influence of factors released from cancer-associated fibroblasts (CAFs) that induce genome methylation changes required for EMT and stemness in EMT-prone PC3 and DU145 PCa cells [194]. Similarly, a reduction in DNA methyltransferase 1 (DNMT1) may promote induction of EMT and the cancer stem cell (CSC) phenotype, which facilitates tumorigenesis in PCa cells [201].

Similarly to other epigenetic changes, histone modifications (predominately at the N-terminal tail) alter DNA availability to the transcriptional machinery and include processes such as lysine and arginine methylation, arginine citrullination, lysine acetylation, and serine/threonine/tyrosine phosphorylation [202]. Histone acetylation at the N-terminal involves the addition of acetyl groups to lysine residues by acetyltransferases (HATs), promoting euchromatin formation and, therefore, increasing DNA functionality. The removal of acetyl groups by deacetylases (HDACs) leads to chromatin concentration and consequent suppression of tumor suppressor genes [203]. Several epigenetic histone modifiers such as lysine methyltransferases (KMT) and demethylases (KDM), SUV39H1 (KMT1A), and SETDB1 (KMT1E) have been shown to control transcriptional gene regulation and to promote cell migration and invasion in PCa [204,205,206]. For example, increased levels of SETDB1 have been associated with the development and prognosis of bone metastases from PCa, whereas the crucial protein arginine methyltransferase 5 (PRMT5) oncogene has been shown to inactivate several tumor suppressors via arginine methylation at H4R3 histone, thus promoting PCa development [207]. Furthermore, arginine methylation by PRMT5 and its interaction with sp1 increases AR-targeted gene expression. Moreover, members of the histone methyltransferase nuclear receptor binding SET domain (NSD) family of proteins NSD1, NSD2, and NSD3 have been associated with carcinogenesis [208]. In particular, NSD2 overexpression has been reported in a variety of solid tumors, including metastatic PCa (in comparison to primary tumors) and is associated with biochemical recurrence, with in vitro studies in PCa cell lines indicating NSD2’s role in the progression of PCa. NSD2 has also been found to regulate the Twist family bHLH transcription factor 1 (TWIST1), which seems to induce EMT in PCa cell lines [209]. Interestingly, in multiple PCa cell lines, histone phosphorylation of H2AX variant at Ser139 may activate checkpoint proteins for cell-cycle arrest, since it promotes the detection of double-strand breaks (DSB) by key components of DNA damage repair [210]. Mono-ubiquitination of H2A in prostate tumors is reduced in comparison to normal tissues, while mono-ubiquitination of H2B at K120 was found to regulate the self-renewal property of PCa stem cells [210].

The activity expression of enzymes regulating an epigenetic program may be simply reflected in a certain histone mark. Histone modifications and their regulators are attractive targets of therapeutic approaches as they represent a dynamic and potentially reversible epigenetic event [211]. The recent interest for the use of epigenetic drugs (“epidrugs”) such as 5-azacitidine (5-Aza) seems rational, as they offer a promising addition in the armamentarium of agents reversing the EMT-induced resistance to therapy in many tumors, including PCa. However, the complexity of the mechanisms involved is still elusive; for instance, 5-Aza may control the EMT process but simultaneously induces neuroendocrine differentiation in PCa cells via reduction of two histone marks, H3k9me3 and H3k27me3 [212].

Another important player in the epigenetic plasticity affecting the cellular plasticity during the EMT process is the effect of several classes of noncoding RNAs (ncRNAs) in transcription regulation and EMT [213]. These classes mainly include small ncRNAs (<200 nt) and long ncRNAs (lncRNAs), with microRNAs (miRNAs) being the best studied in epithelial–mesenchymal plasticity. MicroRNAs feature 22 noncoding nucleotides integrated into the silencing complex (RNA silencing complex) that suppress gene expression post-transcriptionally by guiding Argonaute (AGO) proteins to target sites in the 3′ untranslated region (UTR) of mRNAs [214]. Several of these miRNAs have been associated with critical regulators of EMT, either as inducers or inhibitors. The miR-200 family (miR-200a, miR-200b, miR-141 and miR-429), for example, has described as an epithelial marker, since its members target the expression of ZEB1 and ZEB2 mRNAs and consequently suppress the mesenchymal transition [213]. MiR-155, on the other hand, is highly expressed in several cancers, and numerous studies have identified the role of miR-155, as well as miR-125b and miR-130b, in the oncogenic reprogramming of PC-recruited stem cells [215]. Furthermore, mechanistic studies using adipose-derived stem cells showed that exosomal trafficking of miR-125b and miR-155 could initiate neoplastic reprogramming via targeted inhibition of the LATS2 (large tumor suppressor homolog2) and PGCD4 (programed cell death protein 4) [215]. Moreover, miR-125 targets proapoptotic genes in PCa, thus acting as an oncogene, and miR-22 silences PTEN, which activates PI3K signaling in PCa cells, contributing to prostate tumorigenesis [216]. Similarly, miR-27a acts as an androgen-regulated oncomir in PCa via targeting the tumor suppressor and AR corepressor Prohibitin (PHB), miR-18 enhances AKT phosphorylation by decreasing the tumor suppressor serine/threonine-protein kinase (STK4) levels in PCa cells, and miR-1, miR-29b, and miR-200 have been associated with EMT, thus contributing to PCa progression [217,218].

Tumor suppressor miRNAs have also been studied extensively in relation to their anti-EMT properties. Mir-1 and miR-200 were found reduced with progression of prostate adenocarcinoma and their forced expression inhibited both EMT (via SLUG repression) and tumorigenesis in human and mouse model systems [219]. The downregulation of miR-29b and miR-130b seems to modulate the extracellular matrix structure via matrix metalloproteinase 2 (MMP2) and, similarly, pathological loss of miR-205, as widely observed in PCa, may favor tumorigenesis by creating discontinuities in the basement membrane [220,221,222]. Moreover, miR-146a expression was found significantly decreased in CRPC tissues, and its ectopic overexpression in androgen-independent cell lines decreased tumorigenicity and angiogenesis in vivo by targeting the EGFR pathway [223]. In a recent study, miR-33a-5p expression was downregulated in PCa tissues with bone metastasis and bone-derived cells, and a double-negative loop of miR-33a-5p with ZEB1 was indicated via TGF-β signaling [224]. These findings are in accordance with older studies in which a similar double-negative feedback loop between ZEB2 and miR-145 was revealed [225].

Long noncoding RNA (lncRNA) is an endogenous transcribed RNA molecule containing 200 to 100,000 nucleotides. A well-known representative is the prostate specific PCa antigen 3 (PCA3/DD3) which holds a key role in EMT through several cancer-related genes encoding EMT markers, for instance, MTA2 and PLAUR [226]. Slug-activated PCA3 is also known to promote PRKD3-mediated invasion and migration of PCa cells [227]. LncRNA VIM-AS1 was found to be overexpressed in PCa tissues and cell lines and promoted PCa proliferation and metastasis via EMT through vimentin regulation [228]. Links with novel EMT regulators have also been revealed; the lncRNA MALAT1 enhances the oncogenic activities of EZH2, acting as a crucial RNA cofactor, in castration-resistant PCa [229]. Additionally, the plasmacytoma variant translocation 1 (PVT1), a novel long noncoding RNA, promotes PCa invasion and metastasis by modulating EMT through upregulation of Twist1 via a sponge effect [230]. Similar sponging effects were shown for the HOXA transcript at the distal tip (HOTTIP) lncRNA [231].

## 4. EMT and Therapy Resistance in Prostate Cancer

**Figure 3:**
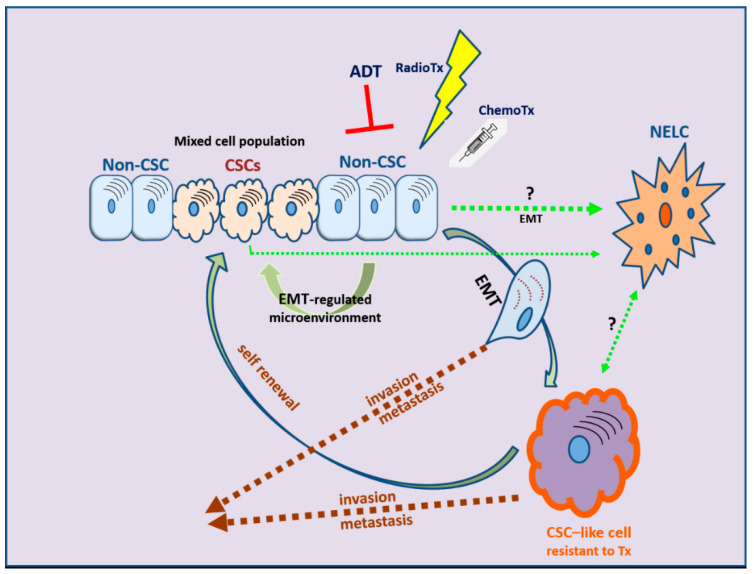
The development of therapy resistance: the complex interlinks among therapeutic manipulations, EMT, and cancer stem cell (CSC) development. NELC: neuro-endocrine-like cell; ADT: androgen deprivation therapy; RadioTx: radiotherapy; ChemoTx: chemotherapy.

EMT is evidently correlated not only with tumor metastasis but, most importantly, also with therapy resistance. The role of EMT is distinct in several tumors, including solid tumors and endocrine-related tumors such as breast and prostate carcinoma [232,233,234,235]. The stem-cell features acquired during the EMT process have revealed that the EMT program is a critical regulator of the cancer stem cell (CSC) phenotype, mainly through epigenetic changes (Figure 3). CSCs are more resistant than non-CSCs to various types of conventional therapies and this may account for treatment failure and CSC-mediated clinical relapse [236]. In preclinical and clinical models, chemotherapy and/or radiotherapy have been shown to eliminate the bulk population of mainly non-CSCs, while leaving behind considerable numbers of CSCs in multiple cancer types [236].

### 4.1. Androgen Deprivation Therapy

The mainstay of advanced or metastatic PCa therapy remains androgen blockade, i.e., ADT. As discussed earlier, the androgen receptor plays a disputable role in the course of the disease, eventually acting as an undesired promoter of EMT, leading to CRPC status. Although solid evidence is still missing, the rationale of combination therapies that aim at both androgen and EMT pathways is intriguing. Hence, dual approaches include the use of TGF-β inhibitors that may suppress both TWIST1 and AR expression or TGF-β receptor inhibitors that exert FOXA1-like suppressor effects on TGF-β (galunisertib), the latter being currently used in a clinical trial in combination with the antiandrogen enzalutamide [49,237,238]. Another approach is focusing on the presence of androgen receptor splice variants or truncated receptors that are often present in CPRC. These aberrant receptors may be inhibited by novel retinamides [239,240]. In addition to retinoids, the use of novel AR antagonists that target both wild-type and mutated ligand-binding domain variants to inhibit AR nuclear translocation has led to favorable results both in vitro and in vivo [241]. Last but not least, the neuroendocrine differentiation (NED) eventually induced by androgen ablation is an important factor for treatment failure, with the EMT process again playing a central role. MicroRNAs, such as miR-652, are implicated in this process [242]. Lately, small-molecule drugs and miRNA mimics have emerged as practical tool in the regulation of miRNAs and in potentially suppressing drug resistance in PCa [243]. An alternative approach would be to target the Wnt/β-catenin signaling pathway associated with NE differentiation [71].

The EMT process in PCa has also been associated with immune evasion due to upregulation of indoleamine 2,3-expression (IDO1) accompanied by an increased number of immunosuppressive regulatory T cells [244]. Similar findings in bladder cancer, where IDO1 expression can upregulate ZEB2 expression probably through miR-200c signaling, provide a promising novel target against immunosuppression in PCa and other tumor types [245].

### 4.2. Chemotherapy

EMT in PCa is associated with resistance to chemotherapy, orchestrated mainly by the action of transcription factors such as ZEB1 and ZEB2, as well as by downregulation of “pro-epithelial” miRNAs, such as miR-143, miR-145, miR-29b, miR-34b, and the miR-200 family [235,246]. Loss of expression of miR-200 and miR-205c occurs during prolonged treatment with docetaxel, suggesting that their restoration may reinstate chemosensitivity by inhibiting EMT [247]. A different approach has been very recently supported by in vitro results using alternative compounds, such as green tea, flavonoids, and minerals (zinc). Green tea (GT) and quercetin (Q), a flavonoid from apples and onions, enhances the efficacy of docetaxel in androgen-independent PCa cells through multiple mechanisms, including the downregulation of chemoresistance-related proteins [248]. Quercetin was also found to overcome docetaxel resistance in PCa cells via androgen receptor and PI3K/Akt signaling pathways, reversing mesenchymal and stem-like cell changes in phenotype and reducing multidrug resistance (MDR) transporters, such as P-glycoprotein (Pgp) expression [249]. Moreover, quercetin was shown to suppress the EMT process, deactivating the PI3K/Akt signaling pathway and downregulating MALAT1, a long noncoding RNA [250]. Similarly, zinc’s synergistic effect to paclitaxel was demonstrated in PCa cell lines via EMT inhibition by downregulating the expression of TWIST1 [251].

### 4.3. Radiation Therapy

Radiotherapy is an effective treatment option for localized PCa, with favorable response rates; however, cancer cells eventually acquire radioresistance (RR) during fractionated irradiation (IR) [252]. Beyond its established role in tumor invasion, metastasis, and recurrence, EMT is also linked to radioresistance. EMT is closely associated with CRPC, and PCa cells with more mesenchymal markers such as Snail, Vimentin, SOX2, and N-cadherin exhibit radioresistance [253]. The common alterations in PCa cells comprise an increased number of cancer stem cells (CSCs), neuroendocrine differentiation (NED), and epithelial–mesenchymal transition, all of which are interlinked, as discussed earlier. Earlier studies have shown that IR itself may induce EMT in prostate tumors via activation of the cAMP response element-binding protein and cytoplasmic sequestration of the activating transcription factor 2 [254]. Recently, it has be suggested that fractionated irradiation causes an increased expression of pluripotency-associated genes, further inducing CSCs and driving progression of NED, which triggers RR acquisition in PCa [252].

The IR-induced EMT process is mediated by several transcription factors (Snail/Slug, STAT3, Twist, ZEB1, and ZEB2) that are activated by a variety of signaling pathways (i.e., Hedgehog, Notch, TGF-β, Wnt, and others) [255]. Recently, increased EZH2 expression in PCa was associated with metastatic recurrence following external beam radiotherapy [256]. The final mechanisms or radioresistance include prolongation of the production of mitochondrial ROS, which delay cell growth as a mechanism for cell death resistance, modulation of the rate of H_2_O_2_ production and the balance between O_2_^•−^ and H_2_O_2_, and activation of Homeobox B9 for enhancement of DNA damage and repair responses. It has been proposed that radioresistant PCa cells adapt to higher oxidative stress via upregulation of endogenous ROS-generating enzymes and antioxidant proteins [255]. Recently, structural maintenance of chromosome-1 (SMC1A), a subunit of the cohesin complex involved in chromosome cohesion during cell-cycle and DNA repair, was identified as a key factor in acquired radioresistance of metastatic PCa cells. Interestingly, inhibition of SMC1A in a model of DU145 and PC3 cells limited the clonogenic, EMT, and cancer stem-like cell (CSC) properties of cancer cells and rendered them more susceptible to RT [257]. In a similar manner, knockdown of LOXL2, a member of the lysyl oxidase gene family, enhanced radiosensitivity of CRPC cells both in vitro and in vivo, through EMT reversal [258].

### 4.4. The Role of Tumor Microenviroment (TME)

In addition to the PCa cell itself, several signals arising from the microenvironment can play a key role in governing EMT and highly influence cancer progression or clinical outcomes in PCa patients. A great variety of regulators and inducers, through a complex network of intermingled pathways, facilitate the transition to a less differentiated, aggressive phenotype [72]. The key players in the surrounding stroma include vascular and neural networks, fibroblasts (particularly cancer-associated fibroblasts (CAFs)), tumor-associated endothelial cells (TECs), innate and adaptive immune cells, and the altered extracellular matrix [259]. EMT changes are not only guided by the tumor cells; they may be induced by stromal cells, which play a leading role not only in tumorigenesis but also in cancer progression and metastasis. Stromal-induced downregulation of miR-1247 by cancer-associated fibroblasts was shown to promote EMT and increased cell invasion, as well as stemness traits in PCa cells [260]. In a similar manner, adipose stromal cells (ASC) induce epithelial–mesenchymal transition (EMT) in PCa cells, providing a link between obesity-associated EMT and cancer progression. Interestingly, using a hunter-killer peptide D-CAN, previously developed for targeted ASC, a combination therapy with cisplatin was more effective in suppressing growth of mouse PCa allografts and xenografts, even in nonobese mice [261]. Further studies in a genetic model of PCa identified adipose stromal cell-secreted chemocine CXCL12 signaling in prostate epithelium as the main EMT driver and link between obesity and cancer progression [262].

Another paradigm of TME-mediated EMT is the Akt/mTOR pathway. In an EMT mouse model, AP1 was found to mediate EMT and the appearance of disseminated tumor cells via the Akt/mTOR pathway [263]. Although the exact role of the mTOR pathway in PCa remains unclear, previous studies have underlined p-mTOR expression as a factor influencing lymphangiogenesis and lymph node metastasis in PCa patients [264]. A possible link between EMT and the mTOR pathway has been proposed through downregulation of RhoA and Rac1 signaling pathways [265]. Interestingly, the use of novel inhibitors, such as the novel dual mTORC1/C2 inhibitor AZD2014, is able to inhibit migration, invasion, and, more importantly, EMT progression in castration-resistant PCa cell lines, suggesting multiple therapeutic benefits from future mTOR inhibitors [266]. However, the extremely complex and still undeciphered crosstalk of PCa cells with the ever-adapting microenvironment still poses a challenge in understanding and overcoming TME-induced therapy resistance [259,267].

## 5. Conclusions and Future Directions

As in other tumor types, accumulating evidence is emphasizing the role of EMT in PCa progression and metastasis. The prostatic cellular plasticity should be viewed as a flexible process, moving from EMT to MET and vice versa, depending on the tumor cell status and tumor microenvironment. This complex procedure is also implicated in the inevitable resistance to hormone manipulation and radiotherapy, and, at present, conventional therapeutic approaches fail to overcome this problem. A novel approach, aiming at EMT reversal, would focus on three main axes: (a) prevention of EMT induction in early stages, aiming at well-known signaling processes, e.g., TGFβ, (b) selective targeting of cells that have already undergone EMT though blockade of classic EMT markers, and (c) reversal of the EMT process by inducing MET, using differentiation agents such as retinoids and their derivatives. Newer therapeutic approaches using combination therapy by novel anti-androgens/anti-estrogens and chemotherapy may promote MET and lead to clinical benefit. However, the Dr. Jekyll and Mr. Hyde dual nature of combination therapies on EMT should be further elucidated, in order to prevent the trans-differentiation to neuroendocrine tumor type, a common phenomenon that only underlines the highly complex mechanisms of cell plasticity. The role of epigenetic modifications may hopefully shed more light on these mechanisms; using alternative metrics of correlation, several epigenetic regulators, including miRNAs and lncRNAs, have been recently identified as interesting candidates for further studies [268]. The reversible nature of epigenetic modifications and the in vivo stability and favorable pharmacokinetics of small-molecule drugs indicate a promising path for novel diagnostic and therapeutic strategies in PCa.

## References

[1] Bray F., Ferlay J., Soerjomataram I., Siegel R.L., Torre L.A., Jemal A. (2018). Global cancer statistics 2018: GLOBOCAN estimates of incidence and mortality worldwide for 36 cancers in 185 countries. CA Cancer J. Clin..

[2] Torre L.A., Siegel R.L., Ward E.M., Jemal A. (2016). Global Cancer Incidence and Mortality Rates and Trends—An Update. Cancer Epidemiol. Biomark. Prev..

[3] Sartor O. (2020). Why is prostate cancer incidence rising in young men?. Cancer.

[4] Ferlay J., Soerjomataram I., Dikshit R., Eser S., Mathers C., Rebelo M., Parkin D.M., Forman D., Bray F. (2015). Cancer incidence and mortality worldwide: Sources, methods and major patterns in GLOBOCAN 2012. Int. J. Cancer.

[5] Matuszak E.A., Kyprianou N. (2011). Androgen regulation of epithelial–mesenchymal transition in prostate tumorigenesis. Expert Rev. Endocrinol. Metab..

[6] Lamouille S., Xu J., Derynck R. (2014). Molecular mechanisms of epithelial–mesenchymal transition. Nat. Rev. Mol. Cell Biol..

[7] Kalluri R., Weinberg R.A. (2009). The basics of epithelial-mesenchymal transition. J. Clin. Investig..

[8] Thiery J.P., Sleeman J.P. (2006). Complex networks orchestrate epithelial–mesenchymal transitions. Nat. Rev. Mol. Cell Biol..

[9] Thiery J.P., Acloque H., Huang R.Y.-J., Nieto M.A. (2009). Epithelial-Mesenchymal Transitions in Development and Disease. Cell.

[10] Thiery J.P. (2002). Epithelial–mesenchymal transitions in tumour progression. Nat. Rev. Cancer.

[11] Loh C.-Y., Chai J., Tang T., Wong W., Sethi G., Shanmugam M., Chong P., Looi C. (2019). The E-Cadherin and N-Cadherin Switch in Epithelial-to-Mesenchymal Transition: Signaling, Therapeutic Implications, and Challenges. Cells.

[12] Pu H., Begemann D.E., Kyprianou N. (2017). Aberrant TGF-β Signaling Drives Castration-Resistant Prostate Cancer in a Male Mouse Model of Prostate Tumorigenesis. Endocrinology.

[13] Røsland G.V., Dyrstad S.E., Tusubira D., Helwa R., Tan T.Z., Lotsberg M.L., Pettersen I.K.N., Berg A., Kindt C., Hoel F. (2019). Epithelial to mesenchymal transition (EMT) is associated with attenuation of succinate dehydrogenase (SDH) in breast cancer through reduced expression of SDHC. Cancer Metab..

[14] Jia D., Li X., Bocci F., Tripathi S., Deng Y., Jolly M.K., Onuchic J.N., Levine H. (2019). Quantifying Cancer Epithelial-Mesenchymal Plasticity and its Association with Stemness and Immune Response. J. Clin. Med..

[15] De Craene B., Berx G. (2013). Regulatory networks defining EMT during cancer initiation and progression. Nat. Rev. Cancer.

[16] Moreno-Bueno G., Portillo F., Cano A. (2008). Transcriptional regulation of cell polarity in EMT and cancer. Oncogene.

[17] Watanabe K., Villarreal-Ponce A., Sun P., Salmans M.L., Fallahi M., Andersen B., Dai X. (2014). Mammary Morphogenesis and Regeneration Require the Inhibition of EMT at Terminal End Buds by Ovol2 Transcriptional Repressor. Dev. Cell.

[18] Li C., Hong T., Nie Q. (2016). Quantifying the landscape and kinetic paths for epithelial-mesenchymal transition from a core circuit. Phys. Chem..

[19] Li C., Wang J. (2015). Quantifying the Landscape for Development and Cancer from a Core Cancer Stem Cell Circuit. Cancer Res..

[20] Boareto M., Jolly M.K., Goldman A., Pietilä M., Mani S., Sengupta S., Ben-Jacob E., Levine H., Onuchic J.N. (2016). Notch-Jagged signalling can give rise to clusters of cells exhibiting a hybrid epithelial/mesenchymal phenotype. J. R. Soc. Interface.

[21] Bocci F., Jolly M.K., Tripathi S.C., Aguilar M., Hanash S.M., Levine H., Onuchic J.N. (2017). Numb prevents a complete epithelial–mesenchymal transition by modulating Notch signalling. J. R. Soc. Interface.

[22] Hasegawa S., Nagano H., Konno M., Eguchi H., Tomokuni A., Tomimaru Y., Asaoka T., Wada H., Hama N., Kawamoto K. (2016). A crucial epithelial to mesenchymal transition regulator, Sox4/Ezh2 axis is closely related to the clinical outcome in pancreatic cancer patients. Int. J. Oncol..

[23] Thompson E.W., Newgreen D.F., Tarin D. (2005). Carcinoma Invasion and Metastasis: A Role for Epithelial-Mesenchymal Transition?. Cancer Res..

[24] Tsai J.H., Yang J. (2013). Epithelial-mesenchymal plasticity in carcinoma metastasis. Genes Dev..

[25] Liao T.-T., Yang M.-H. (2020). Hybrid Epithelial/Mesenchymal State in Cancer Metastasis: Clinical Significance and Regulatory Mechanisms. Cells.

[26] Kolijn K., Verhoef E., van Leenders G.J. (2015). Morphological and immunohistochemical identification of epithelial-to-mesenchymal transition in clinical prostate cancer. Oncotarget.

[27] Morris S.M., Carter K.T., Baek J.Y., Koszarek A., Yeh M.M., Knoblaugh S.E., Grady W.M. (2015). TGF-β signaling alters the pattern of liver tumorigenesis induced by Pten inactivation. Oncogene.

[28] Horie Y., Suzuki A., Kataoka E., Sasaki T., Hamada K., Sasaki J., Mizuno K., Hasegawa G., Kishimoto H., Iizuka M. (2004). Hepatocyte-specific Pten deficiency results in steatohepatitis and hepatocellular carcinomas. J. Clin. Investig..

[29] Attisano L., Wrana J.L. (2013). Signal integration in TGF-β, WNT, and Hippo pathways. F1000Prime Rep..

[30] Ikushima H., Miyazono K. (2010). TGFβ signalling: A complex web in cancer progression. Nat. Rev. Cancer.

[31] Derynck R., Akhurst R.J., Balmain A. (2001). TGF-β signaling in tumor suppression and cancer progression. Nat. Genet..

[32] Kiyono K., Suzuki H.I., Morishita Y., Komuro A., Iwata C., Yashiro M., Hirakawa K., Kano M.R., Miyazono K. (2009). c-Ski overexpression promotes tumor growth and angiogenesis through inhibition of transforming growth factor-β signaling in diffuse-type gastric carcinoma. Cancer Sci..

[33] Komuro A., Yashiro M., Iwata C., Morishita Y., Johansson E., Matsumoto Y., Watanabe A., Aburatani H., Miyoshi H., Kiyono K. (2009). Diffuse-Type Gastric Carcinoma: Progression, Angiogenesis, and Transforming Growth Factor β Signaling. J. Natl. Cancer Inst..

[34] Roberts A.B., Wakefield L. (2003). The two faces of transforming growth factor in carcinogenesis. Proc. Natl. Acad. Sci. USA.

[35] Massagué J. (1998). TGF-β Signal Transduction. Annu. Rev. Biochem..

[36] Liu S., Chen S., Zeng J. (2018). TGF-β signaling: A complex role in tumorigenesis (Review). Mol. Med. Rep..

[37] Yu Q., Stamenkovic I. (2000). Cell surface localized matrix metalloproteinase 9 proteolytically activates TGF beta and promotes tu-mor invasion and angiogenesis. Genes Dev..

[38] Yu J.S.L., Ramasamy T.S., Murphy N., Holt M., Czapiewski R., Wei S.-K., Cui W. (2015). PI3K/mTORC2 regulates TGF-β/Activin signalling by modulating Smad2/3 activity via linker phosphorylation. Nat. Commun..

[39] Vo B.T., Morton D., Komaragiri S., Millena A.C., Leath C., Khan S.A. (2013). TGF-β Effects on Prostate Cancer Cell Migration and Invasion Are Mediated by PGE2 through Activation of PI3K/AKT/mTOR Pathway. Endocrinology.

[40] Singha P.K., Pandeswara S., Geng H., Lan R., Venkatachalam M.A., Saikumar P. (2014). TGF-β induced TMEPAI/PMEPA1 inhibits canonical Smad signaling through R-Smad sequestration and promotes non-canonical PI3K/Akt signaling by reducing PTEN in triple negative breast cancer. Genes Cancer.

[41] Xie F., Liu J., Li C., Zhao Y. (2016). Simvastatin blocks TGF-β1-induced epithelial-mesenchymal transition in human prostate cancer cells. Oncol. Lett..

[42] Jin H., He Y., Zhao P., Hu Y., Tao J., Chen J., Huang Y. (2019). Targeting lipid metabolism to overcome EMT-associated drug resistance via integrin β3/FAK pathway and tumor-associated macrophage repolarization using legumain-activatable delivery. Theranostics.

[43] Odero-Marah V., Hawsawi O., Henderson V., Sweeney J., Schatten H. (2018). Epithelial-Mesenchymal Transition (EMT) and Prostate Cancer. Cell & Molecular Biology of Prostate Cancer.

[44] Shiao S.L., Chu G.C.-Y., Chung L.W. (2016). Regulation of prostate cancer progression by the tumor microenvironment. Cancer Lett..

[45] Nguyen D.P., Li J., Yadav S.S., Tewari A.K. (2014). Recent insights into NF-κB signalling pathways and the link between inflammation and prostate cancer. BJU Int..

[46] Zhang Q., Helfand B.T., Jang T.L., Zhu L.J., Chen L., Yang X.J., Kozlowski J., Smith N., Kundu S.D., Yang G. (2009). Nuclear Factor-κB-Mediated Transforming Growth Factor-β-Induced Expression of Vimentin Is an Independent Predictor of Biochemical Recurrence after Radical Prostatectomy. Clin. Cancer Res..

[47] Locke J.A., Guns E.S., Lubik A.A., Adomat H.H., Hendy S.C., Wood C.A., Ettinger S.L., Gleave M.E., Nelson C.C. (2008). Androgen Levels Increase by Intratumoral De novo Steroidogenesis during Progression of Castration-Resistant Prostate Cancer. Cancer Res..

[48] Ye H., Cheng J., Tang Y., Liu Z., Xu C., Liu Y., Sun Y. (2012). Human Bone Marrow-Derived Mesenchymal Stem Cells produced TGFbeta Contributes to Progression and Metastasis of Prostate Cancer. Cancer Investig..

[49] Song B., Park S.-H., Zhao J.C., Fong K.-W., Li S., Lee Y., Yang Y.A., Sridhar S., Lu X., Abdulkadir S.A. (2019). Targeting FOXA1-mediated repression of TGF-β signaling suppresses castration-resistant prostate cancer progression. J. Clin. Investig..

[50] Haque S., Morris J.C. (2017). Transforming growth factor-β: A therapeutic target for cancer. Hum. Vaccines Immunother..

[51] Yoon G., Kim J.Y., Choi Y.K., Won Y.S., Lim I.K. (2006). Direct activation of TGF-β1 transcription by androgen and androgen receptor complex in Huh7 human hepatoma cells and its tumor in nude mice. J. Cell. Biochem..

[52] Wang H., Song K., Sponseller T.L., Danielpour D. (2005). Novel Function of Androgen Receptor-associated Protein 55/Hic-5 as a Negative Regulator of Smad3 Signaling. J. Biol. Chem..

[53] Song K., Wang H., Krebs T.L., Wang B., Kelley T.J., Danielpour D. (2010). DHT Selectively Reverses Smad3-Mediated/TGF-β-Induced Responses through Transcriptional Down-Regulation of Smad3 in Prostate Epithelial Cells. Mol. Endocrinol..

[54] Qi W., Gao S., Chu J., Zhou L., Wang Z. (2012). Negative Androgen-Response Elements Mediate Androgen-Dependent Transcriptional Inhibition of TGF- 1 and CDK2 Promoters in the Prostate Gland. J. Androl..

[55] Schaeffer E.M., Marchionni L., Huang Z., Simons B., Blackman A., Yu W., Parmigiani G., Berman D.M. (2008). Androgen-induced programs for prostate epithelial growth and invasion arise in embryogenesis and are reactivated in cancer. Oncogene.

[56] Harris W.P., Mostaghel E.A., Nelson P.S., Montgomery B. (2009). Androgen deprivation therapy: Progress in understanding mechanisms of resistance and optimizing androgen depletion. Nat. Clin. Pract. Urol..

[57] Nieto M., Finn S., Loda M., Hahn W.C. (2007). Prostate cancer: Re-focusing on androgen receptor signaling. Int. J. Biochem. Cell Biol..

[58] Zhu M., Kyprianou N. (2010). Role of androgens and the androgen receptor in epithelial-mesenchymal transition and invasion of prostate cancer cells. FASEB J..

[59] Liu Y.-N., Liu Y., Lee H.-J., Hsu Y.-H., Chen J.-H. (2008). Activated Androgen Receptor Downregulates E-Cadherin Gene Expression and Promotes Tumor Metastasis. Mol. Cell. Biol..

[60] Zhu S., Zhao D., Li C., Li Q., Jiang W., Liu Q., Wang R., Fazli L., Li Y., Zhang L. (2020). BMI1 is directly regulated by androgen receptor to promote castration-resistance in prostate cancer. Oncogene.

[61] Sun Y., Wang B.-E., Leong K.G., Yue P., Li L., Jhunjhunwala S., Chen D., Seo K., Modrusan Z., Gao W.-Q. (2012). Androgen Deprivation Causes Epithelial–Mesenchymal Transition in the Prostate: Implications for Androgen-Deprivation Therapy. Cancer Res..

[62] Jennbacken K., Tešan T., Wang W., Gustavsson H., Damber J.-E., Welén K. (2010). N-cadherin increases after androgen deprivation and is associated with metastasis in prostate cancer. Endocr. Relat. Cancer.

[63] Tanaka H., Kono E., Tran C.P., Miyazaki H., Yamashiro J., Shimomura T., Fazli L., Wada R., Huang J., Vessella R.L. (2010). Monoclonal antibody targeting of N-cadherin inhibits prostate cancer growth, metastasis and castration resistance. Nat. Med..

[64] Li H., Price D.K., Figg W.D. (2007). ADH1, an N-cadherin inhibitor, evaluated in preclinical models of angiogenesis and androgen-independent prostate cancer. Anti-Cancer Drugs.

[65] Jaggi M., Nazemi T., Abrahams N.A., Baker J.J., Galich A., Smith L.M., Balaji K. (2006). N-cadherin switching occurs in high Gleason grade prostate cancer. Prostate.

[66] Blaschuk O.W., Devemy E. (2009). Cadherins as novel targets for anti-cancer therapy. Eur. J. Pharmacol..

[67] Tsai Y.-C., Chen W.-Y., Abou-Kheir W., Zeng T., Yin J.J., Bahmad H., Lee Y.-C., Liu Y.-N. (2018). Androgen deprivation therapy-induced epithelial-mesenchymal transition of prostate cancer through downregulating SPDEF and activating CCL2. Biochim. Biophys. Acta Mol. Basis Dis..

[68] Parimi V., Goyal R., Poropatich K., Yang X.J. (2014). Neuroendocrine differentiation of prostate cancer: A review. Am. J. Clin. Exp. Urol..

[69] Dicken H., Hensley P., Kyprianou N. (2019). Prostate tumor neuroendocrine differentiation via EMT: The road less traveled. Asian J. Urol..

[70] Tiwari R., Manzar N., Bhatia V., Yadav A., Nengroo M.A., Datta D., Carskadon S., Gupta N., Sigouros M., Khani F. (2020). Androgen deprivation upregulates SPINK1 expression and potentiates cellular plasticity in prostate cancer. Nat. Commun..

[71] Yeh Y., Guo Q., Connelly Z., Cheng S., Yang S., Prieto-Domínguez N., Yu X. (2019). Wnt/Beta-Catenin Signaling and Prostate Cancer Therapy Resistance. Adv. Exp. Med. Biol..

[72] Montanari M., Rossetti S., Cavaliere C., D’Aniello C., Malzone M.G., Vanacore D., di Franco R., La Mantia E., Iovane G., Piscitelli R. (2017). Epithelial-mesenchymal transition in prostate cancer: An overview. Oncotarget.

[73] Di Zazzo E., Galasso G., Giovannelli P., di Donato M., Bilancio A., Perillo B., Sinisi A.A., Migliaccio A., Castoria G. (2019). Estrogen Receptors in Epithelial-Mesenchymal Transition of Prostate Cancer. Cancers.

[74] Gyftopoulos K., Sotiropoulou G., Varakis I., Barbalias G. (2000). Cellular Distribution of Retinoic Acid Receptor–α in Benign Hyperplastic and Malignant Human Prostates: Comparison with Androgen, Estrogen and Progesterone Receptor Status. Eur. Urol..

[75] Bonkhoff H., Fixemer T., Hunsicker I., Remberger K. (1999). Estrogen Receptor Expression in Prostate Cancer and Premalignant Prostatic Lesions. Am. J. Pathol..

[76] Bonkhoff H. (2018). Estrogen receptor signaling in prostate cancer: Implications for carcinogenesis and tumor progression. Prostate.

[77] Chakravarty D., Sboner A., Nair S.S., Giannopoulou E.G., Li R., Hennig S., Mosquera J.M., Pauwels J., Park K., Kossai M. (2014). The oestrogen receptor alpha-regulated lncRNA NEAT1 is a critical modulator of prostate cancer. Nat. Commun..

[78] Shen Y., Cao J., Liang Z., Lin Q., Wang J., Yang X., Zhang R., Zong J., Du X., Peng Y. (2019). Estrogen receptor α-NOTCH1 axis enhances basal stem-like cells and epithelial-mesenchymal transition phenotypes in prostate cancer. Cell Commun. Signal..

[79] Wang L., Yang X., Chang Y.W.Y., Qi M., Zhou Z., Zhang J., Han B. (2013). SOX4 is associated with poor prognosis in prostate cancer and promotes epithelial–mesenchymal transition in vitro. Prostate Cancer Prostatic Dis..

[80] Dey P., Velazquez-Villegas L.A., Faria M., Turner A., Jonsson P., Webb P., Williams C., Gustafsson J.-Å., Ström A.M. (2015). Estrogen Receptor β2 Induces Hypoxia Signature of Gene Expression by Stabilizing HIF-1α in Prostate Cancer. PLoS ONE.

[81] Gadkar S., Nair S., Patil S., Kalamani S., Bandivdekar A., Patel V., Chaudhari U., Sachdeva G. (2019). Membrane-initiated estrogen signaling in prostate cancer: A route to epithelial-to-mesenchymal transition. Mol. Carcinog..

[82] Lafront C., Germain L., Weidmann C., Audet-Walsh É. (2020). A Systematic Study of the Impact of Estrogens and Selective Estrogen Receptor Modulators on Prostate Cancer Cell Proliferation. Sci. Rep..

[83] Varkaris A., Katsiampoura A.D., Araujo J.C., Gallick G.E., Corn P.G. (2014). Src signaling pathways in prostate cancer. Cancer Metastasis Rev..

[84] Thomas S.M., Brugge J.S. (1997). Cellular Functions Regulated by Src Family Kinases. Annu. Rev. Cell Dev. Biol..

[85] Marzia M., Sims N.A., Voit S., Migliaccio S., Taranta A., Bernardini S., Faraggiana T., Yoneda T., Mundy G.R., Boyce B.F. (2000). Decreased C-Src Expression Enhances Osteoblast Differentiation and Bone Formation. J. Cell Biol..

[86] Roskoski R. (2004). Src protein–tyrosine kinase structure and regulation. Biochem. Biophys. Res. Commun..

[87] Manetti F., Botta M. (2007). Src Inhibitors and Angiogenesis. Curr. Pharm. Des..

[88] Guarino M. (2009). Src signaling in cancer invasion. J. Cell. Physiol..

[89] Fizazi K. (2007). The role of Src in prostate cancer. Ann. Oncol..

[90] Gelman I.H. (2014). Androgen Receptor Activation in Castration-Recurrent Prostate Cancer: The Role of Src-Family and Ack1 Tyrosine Kinases. Int. J. Biol. Sci..

[91] Dai Y., Siemann D. (2019). c-Src is required for hypoxia-induced metastasis-associated functions in prostate cancer cells. OncoTargets Ther..

[92] Lu Y., Dong B., Xu F., Xu Y., Pan J., Song J., Zhang J., Huang Y., Xue W. (2019). CXCL1-LCN2 paracrine axis promotes progression of prostate cancer via the Src activation and epithelial-mesenchymal transition. Cell Commun. Signal..

[93] Alwanian W.M., Tyner A.L. (2020). Protein tyrosine kinase 6 signaling in prostate cancer. Am. J. Clin. Exp. Urol..

[94] Baron S., Manin M., Beaudoin C., Leotoing L., Communal Y., Veyssiere G., Morel L. (2004). Androgen Receptor Mediates Non-genomic Activation of Phosphatidylinositol 3-OH Kinase in Androgen-sensitive Epithelial Cells. J. Biol. Chem..

[95] Zamagni A., Cortesi M., Zanoni M., Tesei A. (2019). Non-nuclear AR Signaling in Prostate Cancer. Front. Chem..

[96] Liu P., Wakamiya M., Shea M.J., Albrecht U., Behringer R.R., Bradley A. (1999). Requirement for Wnt3 in vertebrate axis formation. Nat. Genet..

[97] Popperl H., Schmidt C., Wilson V., Hume C.R., Dodd J., Krumlauf R., Beddington R.S. (1997). Misexpression of Wnt8C in the mouse induces an ectopic embry-onic axis and causes a truncation of the anterior neuroectoderm. Development.

[98] Garcia-Castro M.I., Marcelle C., Bronner-Fraser M. (2002). Ectodermal Wnt function as a neural crest inducer. Science.

[99] Carmona-Fontaine C., Matthews H.K., Kuriyama S., Moreno M., Dunn G.A., Parsons M., Stern C.D., Mayor R. (2008). Contact inhibition of locomotion in vivo controls neural crest directional migration. Nature.

[100] Brabletz T., Jung A., Reu S., Porzner M., Hlubek F., Kunz-Schughart L.A., Knuechel R., Kirchner T. (2001). Variable -catenin expression in colorectal cancers indicates tumor progression driven by the tumor environment. Proc. Natl. Acad. Sci. USA.

[101] Yilmaz M., Christofori G. (2009). EMT, the cytoskeleton, and cancer cell invasion. Cancer Metastasis Rev..

[102] Robinson D.R., Zylstra C.R., Williams B. (2008). Wnt Signaling and Prostate Cancer. Curr. Drug Targets.

[103] Li Y., Wang L., Zhang M., Melamed J., Liu X., Reiter R., Wei J., Peng Y., Zou X., Pellicer A. (2009). LEF1 in Androgen-Independent Prostate Cancer: Regulation of Androgen Receptor Expression, Prostate Cancer Growth, and Invasion. Cancer Res..

[104] Wang G., Wang J., Sadar M.D. (2008). Crosstalk between the Androgen Receptor and β-Catenin in Castrate-Resistant Prostate Cancer. Cancer Res..

[105] Schmalhofer O., Brabletz S., Brabletz T. (2009). E-cadherin, β-catenin, and ZEB1 in malignant progression of cancer. Cancer Metastasis Rev..

[106] Polette M., Mestdagt M., Bindels S., Nawrocki-Raby B., Hunziker W., Foidart J.-M., Birembaut P., Gilles C. (2007). β-Catenin and ZO-1: Shuttle Molecules Involved in Tumor Invasion-Associated Epithelial-Mesenchymal Transition Processes. Cells Tissues Organs.

[107] Whitaker H.C., Girling J., Warren A.Y., Leung H., Mills I.G., Neal D.E. (2008). Alterations in β-catenin expression and localization in prostate cancer. Prostate.

[108] Saha B., Arase A., Imam S.S., Tsao-Wei D., Naritoku W.Y., Groshen S., Jones L.W. (2008). Overexpression of E-cadherin and β-Catenin proteins in metastatic prostate cancer cells in bone. Prostate.

[109] Li Q., Xia D., Wang Z., Liu B., Zhang J., Peng P., Tang Q., Dong J., Guo J., Kuang D. (2021). Circadian Rhythm Gene PER3 Negatively Regulates Stemness of Prostate Cancer Stem Cells via WNT/β-Catenin Signaling in Tumor Microenvironment. Front. Cell Dev. Biol..

[110] Khan A.Q., Ahmed E.I., Elareer N.R., Junejo K., Steinhoff M., Uddin S. (2019). Role of miRNA-Regulated Cancer Stem Cells in the Pathogenesis of Human Malignancies. Cells.

[111] Briscoe J., Therond P. (2013). The mechanisms of Hedgehog signalling and its roles in development and disease. Nat. Rev. Mol. Cell Biol..

[112] Chiang C., Litingtung Y., Lee E., Young K.E., Corden J.L., Westphal H., Beachy P.A. (1996). Cyclopia and defective axial patterning in mice lacking Sonic hedgehog gene function. Nature.

[113] Monsoro-Burq A.-H. (2005). Sclerotome development and morphogenesis: When experimental embryology meets genetics. Int. J. Dev. Biol..

[114] Li X., Deng W., Nail C.D., Bailey S.K., Kraus M.H., Ruppert J.M., Lobo-Ruppert S.M. (2006). Snail induction is an early response to Gli1 that determines the efficiency of epithelial transformation. Oncogene.

[115] Gonnissen A., Isebaert S., Haustermans K. (2013). Hedgehog Signaling in Prostate Cancer and Its Therapeutic Implication. Int. J. Mol. Sci..

[116] Fendrich V., Waldmann J., Esni F., Ramaswamy A., Mullendore M., Buchholz M., Maitra A., Feldmann G. (2007). Snail and Sonic Hedgehog activation in neuroendocrine tumors of the ileum. Endocr. Relat. Cancer.

[117] Yauch R.L., Gould S.E., Scales S., Tang T., Tian H., Ahn C.P., Marshall D., Fu L., Januario T., Kallop D. (2008). A paracrine requirement for hedgehog signalling in cancer. Nature.

[118] Tian H., Callahan C.A., DuPree K.J., Darbonne W.C., Ahn C.P., Scales S.J., de Sauvage F.J. (2009). Hedgehog signaling is restricted to the stromal compartment during pancreatic carcinogenesis. Proc. Natl. Acad. Sci. USA.

[119] Fan L., Pepicelli C.V., Dibble C.C., Catbagan W., Zarycki J.L., Laciak R., Gipp J., Shaw A., Lamm M.L.G., Munoz A. (2004). Hedgehog Signaling Promotes Prostate Xenograft Tumor Growth. Endocrinology.

[120] Scales S., de Sauvage F.J. (2009). Mechanisms of Hedgehog pathway activation in cancer and implications for therapy. Trends Pharmacol. Sci..

[121] Sheng T., Li C., Zhang X., Chi S., He N., Chen K., McCormick F., Gatalica Z., Xie J. (2004). Activation of the hedgehog pathway in advanced prostate cancer. Mol. Cancer.

[122] Datta S., Datta M.W. (2006). Sonic Hedgehog signaling in advanced prostate cancer. Cell. Mol. Life Sci..

[123] Sanchez P., Hernández A.M., Stecca B., Kahler A.J., deGueme A.M., Barrett A., Beyna M., Datta M.W., Datta S., i Altaba A.R. (2004). Inhibition of prostate cancer proliferation by interference with Sonic Hedgehog-GLI1 signaling. Proc. Natl. Acad. Sci. USA.

[124] Tzelepi V., Karlou M., Wen S., Hoang A., Logothetis C., Troncoso P., Efstathiou E. (2011). Expression of hedgehog pathway components in prostate carcinoma microenvironment: Shifting the balance towards autocrine signalling. Histopathology.

[125] Azoulay S., Terry S., Chimingqi M., Sirab N., Faucon H., Medina S.G.D.D., Moutereau S., Maillé P., Soyeux P., Abbou C. (2008). Comparative expression of Hedgehog ligands at different stages of prostate carcinoma progression. J. Pathol..

[126] Kim T.-J., Lee J.Y., Hwang T.-K., Kang C.S., Choi Y.-J. (2011). Hedgehog signaling protein expression and its association with prognostic parameters in prostate cancer: A retrospective study from the view point of new 2010 anatomic stage/prognostic groups. J. Surg. Oncol..

[127] Lubik A.A., Nouri M., Truong S., Ghaffari M., Adomat H.H., Corey E., Cox M.E., Li N., Guns E.S., Yenki P. (2017). Paracrine sonic hedgehog signaling contributes significantly to acquired steroidogenesis in the prostate tumor microenvironment. Int. J. Cancer.

[128] Ishii A., Shigemura K., Kitagawa K., Sung S.-Y., Chen K.-C., Yi-Te C., Liu M.-C., Fujisawa M. (2020). Anti-tumor Effect of Hedgehog Signaling Inhibitor, Vismodegib, on Castration-resistant Prostate Cancer. Anticancer. Res..

[129] Su Q., Xin L. (2016). Notch signaling in prostate cancer: Refining a therapeutic opportunity. Histol. Histopathol..

[130] Deng G., Ma L., Meng Q., Ju X., Jiang K., Jiang P., Yu Z. (2015). Notch signaling in the prostate: Critical roles during development and in the hallmarks of prostate cancer biology. J. Cancer Res. Clin. Oncol..

[131] Leong K.G., Gao W.-Q. (2008). The Notch pathway in prostate development and cancer. Differentiation.

[132] Zhang L., Sha J., Yang G., Huang X., Bo J., Huang Y. (2017). Activation of Notch pathway is linked with epithelial-mesenchymal transition in prostate cancer cells. Cell Cycle.

[133] Zhu H., Zhou X., Redfield S., Lewin J., Miele L. (2013). Elevated Jagged-1 and Notch-1 expression in high grade and metastatic prostate cancers. Am. J. Transl. Res..

[134] Zhang J., Kuang Y., Wang Y., Xu Q., Ren Q. (2017). Notch-4 silencing inhibits prostate cancer growth and EMT via the NF-κB pathway. Apoptosis.

[135] Day K.C., Hiles G.L., Kozminsky M., Dawsey S.J., Paul A., Broses L.J., Shah R., Kunja L.P., Hall C., Palanisamy N. (2017). HER2 and EGFR Overexpression Support Metastatic Progression of Prostate Cancer to Bone. Cancer Res..

[136] Hardbower D.M., Coburn L.A., Asim M., Singh K., Sierra J.C., Barry D.P., Gobert A.P., Piazuelo M.B., Washington M.K., Wilson K.T. (2017). EGFR-mediated macrophage activation promotes colitis-associated tumorigenesis. Oncogene.

[137] Jia Y., Yun C.-H., Park E., Ercan D., Manuia M., Juarez J., Xu C., Rhee K., Chen T., Zhang H. (2016). Overcoming EGFR(T790M) and EGFR(C797S) resistance with mutant-selective allosteric inhibitors. Nature.

[138] Cargnello M., Roux P.P. (2011). Activation and Function of the MAPKs and Their Substrates, the MAPK-Activated Protein Kinases. Microbiol. Mol. Biol. Rev..

[139] Lo H.-W., Xia W., Wei Y., Ali-Seyed M., Huang S.-F., Hung M.-C. (2005). Novel prognostic value of nuclear epidermal growth factor receptor in breast cancer. Cancer Res..

[140] Marti U., Ruchti C., Kämpf J., Thomas G.A., Williams E.D., Peter H.J., Gerber H., Bürgi U. (2001). Nuclear Localization of Epidermal Growth Factor and Epidermal Growth Factor Receptors in Human Thyroid Tissues. Thyroid.

[141] Psyrri A., Yu Z., Weinberger P.M., Sasaki C., Haffty B., Camp R., Rimm D., Burtness B.A. (2005). Quantitative Determination of Nuclear and Cytoplasmic Epidermal Growth Factor Receptor Expression in Oropharyngeal Squamous Cell Cancer by Using Automated Quantitative Analysis. Clin. Cancer Res..

[142] Dittmann K., Mayer C., Fehrenbacher B., Schaller M., Kehlbach R., Rodemann H.P. (2010). Nuclear EGFR shuttling induced by ionizing radiation is regulated by phosphorylation at residue Thr654. FEBS Lett..

[143] Liccardi G., Hartley J.A., Hochhauser D. (2011). EGFR Nuclear Translocation Modulates DNA Repair following Cisplatin and Ionizing Radiation Treatment. Cancer Res..

[144] Hsu S.-C., Miller S.A., Wang Y., Hung M.-C. (2009). Nuclear EGFR is required for cisplatin resistance and DNA repair. Am. J. Transl. Res..

[145] Salomon D.S., Brandt R., Ciardiello F., Normanno N. (1995). Epidermal growth factor-related peptides and their receptors in human malignancies. Crit. Rev. Oncol. Hematol..

[146] Shah R.B., Ghosh D., Elder J.T. (2006). Epidermal growth factor receptor (ErbB1) expression in prostate cancer progression: Correlation with androgen independence. Prostate.

[147] Di Lorenzo G., Tortora G., d’Armiento F.P., de Rosa G., Staibano S., Autorino R., d’Armiento M., de Laurentiis M., de Placido S., Catalano G. (2002). Expression of epidermal growth factor receptor correlates with disease relapse and progression to androgen-independence in human prostate cancer. Clin. Cancer Res..

[148] Traish A.M., Morgentaler A. (2009). Epidermal growth factor receptor expression escapes androgen regulation in prostate cancer: A potential molecular switch for tumour growth. Br. J. Cancer.

[149] Frawley T., Piskareva O. (2020). Extracellular Vesicle Dissemination of Epidermal Growth Factor Receptor and Ligands and Its Role in Cancer Progression. Cancers.

[150] Liao Y., Guo Z., Xia X., Liu Y., Huang C., Jiang L., Wang X., Liu J., Huang H. (2019). Inhibition of EGFR signaling with Spautin-1 represents a novel therapeutics for prostate cancer. J. Exp. Clin. Cancer Res..

[151] Correa R.J., Valdes Y.R., Peart T.M., Fazio E.N., Bertrand M., McGee J., Préfontaine M., Sugimoto A., DiMattia G.E., Shepherd T.G. (2014). Combination of AKT inhibition with autophagy blockade effectively reduces ascites-derived ovarian cancer cell viability. Carcinogenesis.

[152] Horie R., Nakamura O., Yamagami Y., Mori M., Nishimura H., Fukuoka N., Yamamoto T. (2016). Apoptosis and antitumor effects induced by the combination of an mTOR inhibitor and an autophagy inhibitor in human osteosarcoma MG63 cells. Int. J. Oncol..

[153] Shao S., Li S., Qin Y., Wang X., Yang Y., Bai H., Zhou L., Zhao C., Wang C. (2014). Spautin-1, a novel autophagy inhibitor, enhances imatinib-induced apoptosis in chronic myeloid leukemia. Int. J. Oncol..

[154] Bach D.-H., Long N.P., Luu T.-T.-T., Anh N.H., Kwon S.W., Lee S.K. (2018). The Dominant Role of Forkhead Box Proteins in Cancer. Int. J. Mol. Sci..

[155] Wang T., Zheng L., Wang Q., Hu Y.-W. (2018). Emerging roles and mechanisms of FOXC2 in cancer. Clin. Chim. Acta.

[156] Hollier B.G., Tinnirello A.A., Werden S.J., Evans K.W., Taube J., Sarkar T.R., Sphyris N., Shariati M., Kumar S.V., Battula V.L. (2013). FOXC2 Expression Links Epithelial–Mesenchymal Transition and Stem Cell Properties in Breast Cancer. Cancer Res..

[157] Li C., Ding H., Tian J., Wu L., Wang Y., Xing Y., Chen M. (2016). Forkhead Box Protein C2 Promotes Epithelial-Mesenchymal Transition, Migration and Invasion in Cisplatin-Resistant Human Ovarian Cancer Cell Line (SKOV3/CDDP). Cell. Physiol. Biochem..

[158] Børretzen A., Gravdal K., Haukaas S.A., Beisland C., Akslen L.A., Halvorsen O.J. (2019). FOXC2 expression and epithelial–mesenchymal phenotypes are associated with castration resistance, metastasis and survival in prostate cancer. J. Pathol. Clin. Res..

[159] Paranjape A.N., Soundararajan R., Werden S.J., Joseph R., Taube J., Liu H., Rodriguez-Canales J., Sphyris N., Wistuba I., Miura N. (2016). Inhibition of FOXC2 restores epithelial phenotype and drug sensitivity in prostate cancer cells with stem-cell properties. Oncogene.

[160] Teng M., Zhou S., Cai C., Lupien M., He H.H. (2021). Pioneer of prostate cancer: Past, present and the future of FOXA1. Protein Cell.

[161] Gao S., Chen S., Han D., Wang Z., Li M., Han W., Besschetnova A., Liu M., Zhou F., Barrett D. (2020). Chromatin binding of FOXA1 is promoted by LSD1-mediated demethylation in prostate cancer. Nat. Genet..

[162] Ma Z., Xin Z., Hu W., Jiang S., Yang Z., Yan X., Li X., Yang Y., Chen F. (2018). Forkhead box O proteins: Crucial regulators of cancer EMT. Semin. Cancer Biol..

[163] Alwhaibi A., Verma A., Artham S., Adil M.S., Somanath P.R. (2019). Nodal pathway activation due to Akt1 suppression is a molecular switch for prostate cancer cell epithelial-to-mesenchymal transition and metastasis. Biochem. Pharmacol..

[164] Saad F., Shore N., Zhang T., Sharma S., Cho H.K., Jacobs I.A. (2019). Emerging therapeutic targets for patients with advanced prostate cancer. Cancer Treat. Rev..

[165] Gan L., Xu M., Hua R., Tan C., Zhang J., Gong Y., Wu Z., Weng W., Sheng W., Guo W. (2018). The polycomb group protein EZH2 induces epithelial–mesenchymal transition and pluripotent phenotype of gastric cancer cells by binding to PTEN promoter. J. Hematol. Oncol..

[166] Perotti V., Baldassari P., Molla A., Nicolini G., Bersani I., Grazia G., Benigni F., Maurichi A., Santinami M., Anichini A. (2019). An actionable axis linking NFATc2 to EZH2 controls the EMT-like program of melanoma cells. Oncogene.

[167] Lobo J., Rodrigues Â., Antunes L., Graça I., Ramalho-Carvalho J., Vieira F.Q., Martins A.T., Oliveira J., Jeronimo C., Henrique R. (2018). High immunoexpression of Ki67, EZH2, and SMYD3 in diagnostic prostate biopsies independently predicts outcome in patients with prostate cancer. Urol. Oncol. Semin. Orig. Investig..

[168] Xu K., Wu Z.J., Groner A.C., He H.H., Cai C., Lis R.T., Wu X., Stack E.C., Loda M., Liu T. (2012). EZH2 Oncogenic Activity in Castration-Resistant Prostate Cancer Cells Is Polycomb-Independent. Science.

[169] Yang Y.A., Yu J. (2013). EZH2, an epigenetic driver of prostate cancer. Protein Cell.

[170] Kim J., Lee Y., Lu X., Song B., Fong K.-W., Cao Q., Licht J.D., Zhao J.C., Yu J. (2018). Polycomb- and Methylation-Independent Roles of EZH2 as a Transcription Activator. Cell Rep..

[171] Wang J., He C., Gao P., Wang S., Lv R., Zhou H., Zhou Q., Zhang K., Sun J., Fan C. (2020). HNF1B-mediated repression of SLUG is suppressed by EZH2 in aggressive prostate cancer. Oncogene.

[172] Tao J., Shi L., Huang L., Shi H., Chen H., Wang Y., Wang T. (2017). EZH2 is involved in silencing of WNT5A during epithelial–mesenchymal transition of colon cancer cell line. J. Cancer Res. Clin. Oncol..

[173] Bai Y., Zhang Z., Cheng L., Wang R., Chen X., Kong Y., Feng F., Ahmad N., Li L., Liu X. (2019). Inhibition of enhancer of zeste homolog 2 (EZH2) overcomes enzalutamide resistance in castration-resistant prostate cancer. J. Biol. Chem..

[174] Taplin M.-E., Hussain A., Shah S., Shore N.D., Agrawal M., Clark W., Edenfield W.J., Nordquist L.T., Sartor O.A., Butrynski J.E. (2019). ProSTAR: A phase Ib/II study of CPI-1205, a small molecule inhibitor of EZH2, combined with enzalutamide (E) or abiraterone/prednisone (A/P) in patients with metastatic castration-resistant prostate cancer (mCRPC). J. Clin. Oncol..

[175] Qiu X., Wang W., Li B., Cheng B., Lin K., Bai J., Li H., Yang G. (2019). Targeting Ezh2 could overcome docetaxel resistance in prostate cancer cells. BMC Cancer.

[176] Elsir T., Smits A., Lindström M.S., Nistér M. (2012). Transcription factor PROX1: Its role in development and cancer. Cancer Metastasis Rev..

[177] Lu M.-H., Huang C.-C., Pan M.-R., Chen H.-H., Hung W.-C. (2012). Prospero Homeobox 1 Promotes Epithelial–Mesenchymal Transition in Colon Cancer Cells by Inhibiting E-cadherin via miR-9. Clin. Cancer Res..

[178] Liu Y., Zhang J.-B., Qin Y., Wang W., Wei L., Teng Y., Guo L., Zhang B., Lin Z., Liu J. (2013). PROX1 promotes hepatocellular carcinoma metastasis by way of up-regulating hypoxia-inducible factor 1α expression and protein stability. Hepatology.

[179] Wang B., Huang J., Zhou J., Hui K., Xu S., Fan J., Li L., Wang X., Hsieh J.-T., He D. (2016). DAB2IP regulates EMT and metastasis of prostate cancer through targeting PROX1 transcription and destabilizing HIF1α protein. Cell. Signal..

[180] Nelson J., Bagnato A., Battistini B., Nisen P. (2003). The endothelin axis: Emerging role in cancer. Nat. Rev. Cancer.

[181] McKenzie G.A.G., Hinsley E.E., Hunter K., Lambert D.W. (2014). The endothelin axis in head and neck cancer: A promising therapeutic opportunity?. J. Oral Pathol. Med..

[182] Gu X., Han S., Cui M., Xue J., Ai L., Sun L., Zhu X., Wang Y., Liu C. (2019). Knockdown of endothelin receptor B inhibits the progression of triple-negative breast cancer. Ann. N. Y. Acad. Sci..

[183] Cianfrocca R., Rosanò L., Tocci P., Sestito R., Caprara V., di Castro V., de Maria R., Bagnato A. (2017). Blocking endothelin-1-receptor/β-catenin circuit sensitizes to chemotherapy in colorectal cancer. Cell Death Differ..

[184] Bagnato A., Rosanò L. (2007). Epithelial-Mesenchymal Transition in Ovarian Cancer Progression: A Crucial Role for the Endothelin Axis. Cells Tissues Organs.

[185] Gyftopoulos K., Papanikolaou S., Bravou V., Papadaki H. (2017). The role of the endothelin axis in promoting epithelial to mesenchymal transition and lymph node metastasis in prostate adenocarcinoma. Urol. Ann..

[186] Qi P., Chen M., Zhang L.-X., Song R.-X., He Z.-H., Wang Z.-P. (2015). A Meta-Analysis and Indirect Comparison of Endothelin A Receptor Antagonist for Castration-Resistant Prostate Cancer. PLoS ONE.

[187] Moon H.H., Clines K.L., Cooks M.A., Cialek C.A., Esvelt M.A., Clines G.A. (2019). Castration Determines the Efficacy of ETAR Blockade in a Mouse Model of Prostate Cancer Bone Metastasis. Endocrinology.

[188] Xu K., Liao Y. (2019). Epigenetic regulation of prostate cancer: The theories and the clinical implications. Asian J. Androl..

[189] Jones P.A. (2012). Functions of DNA methylation: Islands, start sites, gene bodies and beyond. Nat. Rev. Genet..

[190] Curradi M., Izzo A., Badaracco G., Landsberger N. (2002). Molecular Mechanisms of Gene Silencing Mediated by DNA Methylation. Mol. Cell. Biol..

[191] Kumar S., Cheng X., Klimasauskas S., Sha M., Posfai J., Roberts R., Wilson G.G. (1994). The DNA (cytosine-5) methyltransferases. Nucleic Acids Res..

[192] Cheung H.H., Lee T.-L., Rennert O.M., Chan W.-Y. (2009). DNA methylation of cancer genome. Birth Defects Res. Part C Embryo Today Rev..

[193] Ikeda Y., Kinoshita T. (2008). DNA demethylation: A lesson from the garden. Chromosomes.

[194] Pistore C., Giannoni E., Colangelo T., Rizzo F., Magnani E., Muccillo L., Giurato G., Mancini M., Rizzo S., Riccardi M. (2017). DNA methylation variations are required for epithelial-to-mesenchymal transition induced by cancer-associated fibroblasts in prostate cancer cells. Oncogene.

[195] Lin P.-C., Giannopoulou E.G., Park K., Mosquera J.M., Sboner A., Tewari A.K., Garraway L.A., Beltran H., Rubin M.A., Elemento O. (2013). Epigenomic Alterations in Localized and Advanced Prostate Cancer. Neoplasia.

[196] Kang G.H., Lee S., Lee H.J., Hwang K.S. (2004). Aberrant CpG island hypermethylation of multiple genes in prostate cancer and prostatic intraepithelial neoplasia. J. Pathol..

[197] Mahon K.L., Qu W., Devaney J.M., Paul C., Castillo L., Wykes R.J., Chatfield M., Boyer M., Stockler M.R., PRIMe Consortium (2014). Methylated Glutathione S-transferase 1 (mGSTP1) is a potential plasma free DNA epigenetic marker of prognosis and response to chemotherapy in castrate-resistant prostate cancer. Br. J. Cancer.

[198] Suzuki H., Freije D., Nusskern D.R., Okami K., Cairns P., Sidransky D., Isaacs W.B., Bova G.S. (1998). Interfocal heterogeneity of PTEN/MMAC1 gene alterations in multiple metastatic prostate cancer tissues. Cancer Res..

[199] Jarrard D.F., Bova G.S., Ewing C.M., Pin S.S., Nguyen S.H., Baylin S.B., Cairns P., Sidransky D., Herman J.G., Isaacs W.B. (1997). Deletional, mutational, and methylation analyses of CDKN2 (p16/MTS1) in primary and metastatic prostate cancer. Genes Chromosom. Cancer.

[200] Pakneshan P., Xing R.H., Rabbani S.A. (2003). Methylation status of uPA promoter as a molecular mechanism regulating prostate cancer invasion and growth in vitro and in vivo. FASEB J..

[201] Lee E., Wang J., Yumoto K., Jung Y., Cackowski F.C., Decker A.M., Li Y., Franceschi R.T., Pienta K.J., Taichman R.S. (2016). DNMT1 Regulates Epithelial-Mesenchymal Transition and Cancer Stem Cells, Which Promotes Prostate Cancer Metastasis. Neoplasia.

[202] Alhamwe B.A., Khalaila R., Wolf J., von Bülow V., Harb H., Alhamdan F., Hii C.S., Prescott S.L., Ferrante A., Renz H. (2018). Histone modifications and their role in epigenetics of atopy and allergic diseases. Allergy Asthma Clin. Immunol..

[203] Harb H., Alhamwe B.A., Garn H., Renz H., Potaczek D.P. (2016). Recent developments in epigenetics of pediatric asthma. Curr. Opin. Pediatr..

[204] Yu T., Wang C., Yang J., Guo Y., Wu Y., Li X. (2017). Metformin inhibits SUV39H1-mediated migration of prostate cancer cells. Oncogene.

[205] Li Q., Li Y., Wang Y., Cui Z., Gong L., Qu Z., Zhong Y., Zhou J., Zhou Y., Gao Y. (2016). Quantitative proteomic study of human prostate cancer cells with different metastatic potentials. Int. J. Oncol..

[206] Askew E.B., Bai S., Parris A.B., Minges J.T., Wilson E.M. (2017). Androgen receptor regulation by histone methyltransferase Suppressor of variegation 3-9 homolog 2 and Melanoma antigen-A11. Mol. Cell. Endocrinol..

[207] Stopa N., Krebs J.E., Shechter D. (2015). The PRMT5 arginine methyltransferase: Many roles in development, cancer and beyond. Cell. Mol. Life Sci..

[208] Li Y., Trojer P., Xu C.-F., Cheung P., Kuo A., Drury W.J., Qiao Q., Neubert T.A., Xu R.-M., Gozani O. (2009). The Target of the NSD Family of Histone Lysine Methyltransferases Depends on the Nature of the Substrate. J. Biol. Chem..

[209] Ezponda T., Popovic R., Shah M.Y., Martinez-Garcia E., Zheng Y., Min D.-J., Will C., Neri A., Kelleher N.L., Yu J. (2013). The histone methyltransferase MMSET/WHSC1 activates TWIST1 to promote an epithelial–mesenchymal transition and invasive properties of prostate cancer. Oncogene.

[210] Crea F., Clermont P.-L., Mai A., Helgason C. (2014). Histone Modifications, Stem Cells and Prostate Cancer. Curr. Pharm. Des..

[211] Cohen I., Poręba E., Kamieniarz K., Schneider R. (2011). Histone Modifiers in Cancer: Friends or Foes?. Genes Cancer.

[212] Lee E., Wang J., Jung Y., Cackowski F.C., Taichman R.S. (2018). Reduction of two histone marks, H3k9me3 and H3k27me3 by epidrug induces neuroendocrine differentiation in prostate cancer. J. Cell. Biochem..

[213] Sun L., Fang J. (2016). Epigenetic regulation of epithelial–mesenchymal transition. Cell. Mol. Life Sci..

[214] Sekhon K., Bucay N., Majid S., Dahiya R., Saini S. (2016). MicroRNAs and epithelial-mesenchymal transition in prostate cancer. Oncotarget.

[215] Elmageed Z.A., Yang Y., Thomas R., Ranjan M., Mondal D., Moroz K., Fang Z., Rezk B.M., Moparty K., Sikka S.C. (2014). Neoplastic Reprogramming of Patient-Derived Adipose Stem Cells by Prostate Cancer Cell-Associated Exosomes. STEM Cells.

[216] Seashols-Williams S.J., Budd W., Clark G.C., Wu Q., Daniel R., Dragoescu E., Zehner Z.E. (2016). miR-9 Acts as an OncomiR in Prostate Cancer through Multiple Pathways That Drive Tumour Progression and Metastasis. PLoS ONE.

[217] Fletcher C.E., Dart D.A., Sita-Lumsden A., Cheng H., Rennie P.S., Bevan C.L. (2012). Androgen-regulated processing of the oncomir MiR-27a, which targets Prohibitin in prostate cancer. Hum. Mol. Genet..

[218] Hsu T.-I., Hsu C.-H., Lee K.-H., Lin J.-T., Chen C.-S., Chang K.-C., Su C.-Y., Hsiao M., Lu P.-J. (2014). MicroRNA-18a is elevated in prostate cancer and promotes tumorigenesis through suppressing STK4 in vitro and in vivo. Oncogenesis.

[219] Liu Y.-N., Yin J.J., Abou-Kheir W., Hynes P.G., Casey O.M., Fang L., Yi M., Stephens R.M., Seng V., Sheppard-Tillman H. (2013). MiR-1 and miR-200 inhibit EMT via Slug-dependent and tumorigenesis via Slug-independent mechanisms. Oncogene.

[220] Steele R., Mott J.L., Ray R.B. (2010). MBP-1 Upregulates miR-29b, Which Represses Mcl-1, Collagens, and Matrix Metalloproteinase-2 in Prostate Cancer Cells. Genes Cancer.

[221] Gandellini P., Folini M., Longoni N., Pennati M., Binda M., Colecchia M., Salvioni R., Supino R., Moretti R., Limonta P. (2009). miR-205 Exerts Tumor-Suppressive Functions in Human Prostate through Down-regulation of Protein Kinase Cε. Cancer Res..

[222] Gandellini P., Profumo V., Casamichele A., Fenderico N., Borrelli S., Petrovich G., Santilli G., Callari M., Colecchia M., Pozzi S. (2012). miR-205 regulates basement membrane deposition in human prostate: Implications for cancer development. Cell Death Differ..

[223] Xu B., Wang N., Wang X., Tong N., Shao N., Tao J., Li P., Niu X., Feng N., Zhang L. (2012). MiR-146a suppresses tumor growth and progression by targeting EGFR pathway and in a p-ERK-dependent manner in castration-resistant prostate cancer. Prostate.

[224] Dai Y., Wu Z., Lang C., Zhang X., He S., Yang Q., Guo W., Lai Y., Du H., Peng X. (2019). Copy number gain of ZEB1 mediates a double-negative feedback loop with miR-33a-5p that regulates EMT and bone metastasis of prostate cancer dependent on TGF-β signaling. Theranostics.

[225] Ren D., Wang M., Guo W., Huang S., Wang Z., Zhao X., Du H., Song L., Peng X. (2014). Double-negative feedback loop between ZEB2 and miR-145 regulates epithelial-mesenchymal transition and stem cell properties in prostate cancer cells. Cell Tissue Res..

[226] Lo U.-G., Lee C.-F., Lee M.-S., Hsieh J.-T. (2017). The Role and Mechanism of Epithelial-to-Mesenchymal Transition in Prostate Cancer Progression. Int. J. Mol. Sci..

[227] He J.-H., Li B.-X., Han Z.-P., Zou M.-X., Wang L., Lv Y.-B., Zhou J.-B., Cao M.-R., Li Y.-G., Zhang J.-Z. (2016). Snail-activated long non-coding RNA PCA3 up-regulates PRKD3 expression by miR-1261 sponging, thereby promotes invasion and migration of prostate cancer cells. Tumor Biol..

[228] Zhang Y., Zhang J., Liang S., Lang G., Liu G., Liu P., Deng X. (2019). Long non-coding RNA VIM-AS1 promotes prostate cancer growth and invasion by regulating epithelial-mesenchymal transition. Off. J. Balk. Union Oncol..

[229] Wang D., Ding L., Wang L., Zhao Y., Sun Z., Karnes R.J., Zhang J., Huang H. (2015). LncRNA MALAT1 enhances oncogenic activities of EZH2 in castration-resistant prostate cancer. Oncotarget.

[230] Chang Z., Cui J., Song Y. (2018). Long noncoding RNA PVT1 promotes EMT via mediating microRNA-186 targeting of Twist1 in prostate cancer. Gene.

[231] Yang B., Gao G., Wang Z., Sun D., Wei X., Ma Y., Ding Y. (2018). Long non-coding RNA HOTTIP promotes prostate cancer cells proliferation and migration by sponging miR-216a-5p. Biosci. Rep..

[232] Jiborn T., Bjartell A., Abrahamsson P.-A. (1998). Neuroendocrine Differentiation in Prostatic Carcinoma During Hormonal Treatment. Urology.

[233] Arumugam T., Ramachandran V., Fournier K.F., Wang H., Marquis L., Abbruzzese J.L., Gallick G.E., Logsdon C.D., McConkey D.J., Choi W. (2009). Epithelial to Mesenchymal Transition Contributes to Drug Resistance in Pancreatic Cancer. Cancer Res..

[234] Smith B.N., Bhowmick N.A. (2016). Role of EMT in Metastasis and Therapy Resistance. J. Clin. Med..

[235] Culig Z. (2019). Epithelial mesenchymal transition and resistance in endocrine-related cancers. Biochim. Biophys. Acta Mol. Cell Res..

[236] Shibue T., Weinberg T.S.R.A. (2017). EMT, CSCs, and drug resistance: The mechanistic link and clinical implications. Nat. Rev. Clin. Oncol..

[237] Shiota M., Itsumi M., Takeuchi A., Imada K., Yokomizo A., Kuruma H., Inokuchi J., Tatsugami K., Uchiumi T., Oda Y. (2015). Crosstalk between epithelial-mesenchymal transition and castration resistance mediated by Twist1/AR signaling in prostate cancer. Endocr. Relat. Cancer.

[238] Paller C., Pu H., Begemann D.E., Wade C.A., Hensley P., Kyprianou N. (2019). TGF-β receptor I inhibitor enhances response to enzalutamide in a pre-clinical model of advanced prostate cancer. Prostate.

[239] Shao C., Yu B., Liu Y. (2019). Androgen receptor splicing variant 7: Beyond being a constitutively active variant. Life Sci..

[240] Ramamurthy V.P., Ramalingam S., Gediya L.K., Njar V.C.O. (2018). The retinamide VNLG -152 inhibits f-AR/AR -V7 and MNK—eIF 4E signaling pathways to suppress EMT and castration-resistant prostate cancer xenograft growth. FEBS J..

[241] Borgmann H., Lallous N., Ozistanbullu D., Beraldi E., Paul N., Dalal K., Fazli L., Haferkamp A., Lejeune P., Cherkasov A. (2018). Moving Towards Precision Urologic Oncology: Targeting Enzalutamide-resistant Prostate Cancer and Mutated Forms of the Androgen Receptor Using the Novel Inhibitor Darolutamide (ODM-201). Eur. Urol..

[242] Nam R.K., Benatar T., Amemiya Y., Wallis C.J., Romero J.M., Tsagaris M., Sherman C., Sugar L., Seth A. (2018). MicroRNA-652 induces NED in LNCaP and EMT in PC3 prostate cancer cells. Oncotarget.

[243] Doldi V., Pennati M., Forte B., Gandellini P., Zaffaroni N. (2016). Dissecting the role of microRNAs in prostate cancer metastasis: Implications for the design of novel therapeutic approaches. Cell. Mol. Life Sci..

[244] Kolijn K., Verhoef E.I., Smid M., Böttcher R., Jenster G.W., Debets R., van Leenders G.J. (2018). Epithelial–Mesenchymal Transition in Human Prostate Cancer Demonstrates Enhanced Immune Evasion Marked by IDO1 Expression. Cancer Res..

[245] Tsai Y.-S., Jou Y.-C., Tsai H.-T., Cheong I.-S., Tzai T.-S. (2019). Indoleamine-2,3-dioxygenase-1 expression predicts poorer survival and up-regulates ZEB2 expression in human early stage bladder cancer. Urol. Oncol. Semin. Orig. Investig..

[246] Li F., Mahato R.I. (2014). MicroRNAs and Drug Resistance in Prostate Cancers. Mol. Pharm..

[247] Puhr M., Hoefer J., Schäfer G., Erb H.H., Oh S.J., Klocker H., Heidegger I., Neuwirt H., Culig Z. (2012). Epithelial-to-Mesenchymal Transition Leads to Docetaxel Resistance in Prostate Cancer and Is Mediated by Reduced Expression of miR-200c and miR-205. Am. J. Pathol..

[248] Wang P., Henning S.M., Heber D., Vadgama J.V. (2015). Sensitization to docetaxel in prostate cancer cells by green tea and quercetin. J. Nutr. Biochem..

[249] Lu X., Yang F., Chen D., Zhao Q., Chen D., Ping H., Xing N. (2020). Quercetin reverses docetaxel resistance in prostate cancer via androgen receptor and PI3K/Akt signaling pathways. Int. J. Biol. Sci..

[250] Lu X., Chen D., Yang F., Xing N. (2020). Quercetin Inhibits Epithelial-to-Mesenchymal Transition (EMT) Process and Promotes Apoptosis in Prostate Cancer via Downregulating lncRNA MALAT1. Cancer Manag. Res..

[251] Xue Y., Ms B.Y., Liu Y., Guo R., Li J., Ms L.Z., Su J., Sun L., Li Y. (2019). Zinc promotes prostate cancer cell chemosensitivity to paclitaxel by inhibiting epithelial-mesenchymal transition and inducing apoptosis. Prostate.

[252] Murata K., Saga R., Monzen S., Tsuruga E., Hasegawa K., Hosokawa Y. (2019). Understanding the mechanism underlying the acquisition of radioresistance in human prostate cancer cells. Oncol. Lett..

[253] Tsao T., Beretov J., Ni J., Bai X., Bucci J., Graham P., Li Y. (2019). Cancer stem cells in prostate cancer radioresistance. Cancer Lett..

[254] Deng X., Elzey B.D., Poulson J.M., Morrison W.B., Ko S.-C., Hahn N.M., Ratliff T.L., Hu C.-D. (2011). Ionizing radiation induces neuroendocrine differentiation of prostate cancer cells in vitro, in vivo and in prostate cancer patients. Am. J. Cancer Res..

[255] Chaiswing L., Weiss H.L., Jayswal R.D., Clair D.K.S., Kyprianou N. (2018). Profiles of Radioresistance Mechanisms in Prostate Cancer. Crit. Rev. Oncog..

[256] Wu X., Scott H., Carlsson S.V., Sjoberg D.D., Cerundolo L., Lilja H., Prevo R., Rieunier G., Macaulay V., Higgins G.S. (2019). Increased EZH2 expression in prostate cancer is associated with metastatic recurrence following external beam radiotherapy. Prostate.

[257] Yadav S., Kowolik C.M., Lin M., Zuro D., Hui S.K., Riggs A., Horne D.A. (2019). SMC1A is associated with radioresistance in prostate cancer and acts by regulating epithelial-mesenchymal transition and cancer stem-like properties. Mol. Carcinog..

[258] Xie P., Yu H., Wang F., Yan F., He X. (2019). Inhibition of LOXL2 Enhances the Radiosensitivity of Castration-Resistant Prostate Cancer Cells Associated with the Reversal of the EMT Process. BioMed Res. Int..

[259] Wu T., Dai Y. (2017). Tumor microenvironment and therapeutic response. Cancer Lett..

[260] Taddei M.L., Cavallini L., Ramazzotti M., Comito G., Pietrovito L., Morandi A., Giannoni E., Raugei G., Chiarugi P. (2019). Stromal-induced downregulation of miR-1247 promotes prostate cancer malignancy. J. Cell. Physiol..

[261] Su F., Ahn S., Saha A., DiGiovanni J., Kolonin M.G. (2019). Adipose stromal cell targeting suppresses prostate cancer epithelial-mesenchymal transition and chemoresistance. Oncogene.

[262] Su F., Daquinag A.C., Ahn S., Saha A., Dai Y., Zhao Z., DiGiovanni J., Kolonin M.G. (2021). Progression of prostate carcinoma is promoted by adipose stromal cell-secreted CXCL12 signaling in prostate epithelium. NPJ Precis. Oncol..

[263] Shi J., Wang L., Zou C., Xia Y., Qin S., Keller E., Mizokami A., Zhang J., Lu Y. (2018). Tumor microenvironment promotes prostate cancer cell dissemination via the Akt/mTOR pathway. Oncotarget.

[264] Lilis I., Giopanou I., Papadaki H., Gyftopoulos K. (2018). The expression of p-mTOR and COUP-TFII correlates with increased lymphangiogenesis and lymph node metastasis in prostate adenocarcinoma. Urol. Oncol. Semin. Orig. Investig..

[265] Chen X., Cheng H., Pan T., Liu Y., Su Y., Ren C., Huang D., Zha X., Liang C. (2015). mTOR regulate EMT through RhoA and Rac1 pathway in prostate cancer. Mol. Carcinog..

[266] Li S., Sheng J., Liu Z., Fan Y., Zhang C., Lv T., Hu S., Jin J., Yu W., Song Y. (2021). Potent antitumour of the mTORC1/2 dual inhibitor AZD2014 in docetaxel-sensitive and docetaxel-resistant castration-resistant prostate cancer cells. J. Cell. Mol. Med..

[267] Desbats M.A., Giacomini I., Prayer-Galetti T., Montopoli M. (2020). Metabolic Plasticity in Chemotherapy Resistance. Front. Oncol..

[268] Ehsani R., Drabløs F. (2020). Enhanced identification of significant regulators of gene expression. BMC Bioinform..

